# Genome sequence of the necrotrophic plant pathogen *Pythium ultimum *reveals original pathogenicity mechanisms and effector repertoire

**DOI:** 10.1186/gb-2010-11-7-r73

**Published:** 2010-07-13

**Authors:** C André Lévesque, Henk Brouwer, Liliana Cano, John P Hamilton, Carson Holt, Edgar Huitema, Sylvain Raffaele, Gregg P Robideau, Marco Thines, Joe Win, Marcelo M Zerillo, Gordon W Beakes, Jeffrey L Boore, Dana Busam, Bernard Dumas, Steve Ferriera, Susan I Fuerstenberg, Claire MM Gachon, Elodie Gaulin, Francine Govers, Laura Grenville-Briggs, Neil Horner, Jessica Hostetler, Rays HY Jiang, Justin Johnson, Theerapong Krajaejun, Haining Lin, Harold JG Meijer, Barry Moore, Paul Morris, Vipaporn Phuntmart, Daniela Puiu, Jyoti Shetty, Jason E Stajich, Sucheta Tripathy, Stephan Wawra, Pieter van West, Brett R Whitty, Pedro M Coutinho, Bernard Henrissat, Frank Martin, Paul D Thomas, Brett M Tyler, Ronald P De Vries, Sophien Kamoun, Mark Yandell, Ned Tisserat, C Robin Buell

**Affiliations:** 1 Agriculture and Agri-Food Canada, 960 Carling Ave, Ottawa, ON, K1A 0C6, Canada; 2 Department of Biology, Carleton University, Ottawa, ON, K1S 5B6, Canada; 3 CBS-KNAW, Fungal Biodiversity Centre, Uppsalalaan 8, Utrecht, 3584 CT, The Netherlands; 4 The Sainsbury Laboratory, Norwich, NR4 7UH, UK; 5 Department of Plant Biology, Michigan State University, East Lansing, MI 48824, USA; 6 Eccles Institute of Human Genetics, University of Utah, 15 North 2030 East, Room 2100, Salt Lake City, UT 84112-5330, USA; 7 Biodiversity and Climate Research Centre, Georg-Voigt-Str 14-16, D-60325, Frankfurt, Germany; 8 Department of Biological Sciences, Insitute of Ecology, Evolution and Diversity, Johann Wolfgang Goethe University, Siesmayerstr. 70, D-60323 Frankfurt, Germany; 9 Department of Bioagricultural Sciences and Pest Management, Colorado State University, Fort Collins, CO 80523-1177, USA; 10 School of Biology, Newcastle University, Newcastle upon Tyne, NE1 7RU, UK; 11 Genome Project Solutions, 1024 Promenade Street, Hercules, CA 94547, USA; 12 J Craig Venter Institute, 9704 Medical Center Dr., Rockville, MD 20850, USA; 13 Surfaces Cellulaires et Signalisation chez les Végétaux, UMR5546 CNRS-Université de Toulouse, 24 chemin de Borde Rouge, BP42617, Auzeville, Castanet-Tolosan, F-31326, France; 14 Scottish Association for Marine Science, Oban, PA37 1QA, UK; 15 Laboratory of Phytopathology, Wageningen University, NL-1-6708 PB, Wageningen, The Netherlands; 16 Centre for BioSystems Genomics (CBSG), PO Box 98, 6700 AB Wageningen, The Netherlands; 17 Institute of Medical Sciences, University of Aberdeen, Foresterhill, Aberdeen, AB25 2ZD, UK; 18 The Broad Institute of MIT and Harvard, Cambridge, MA 02141, USA; 19 Department of Pathology, Faculty of Medicine-Ramathibodi Hospital, Mahidol University, Rama 6 Road, Bangkok, 10400, Thailand; 20 Department of Biological Sciences, Bowling Green State University, Bowling Green, OH 43403, USA; 21 Department of Plant Pathology and Microbiology, University of California, Riverside, CA 92521, USA; 22 Virginia Bioinformatics Institute, Virginia Polytechnic Institute and State University, Washington Street, Blacksburg, VA 24061-0477, USA; 23 Architecture et Fonction des Macromolecules Biologiques, UMR6098, CNRS, Univ. Aix-Marseille I & II, 163 Avenue de Luminy, 13288 Marseille, France; 24 USDA-ARS, 1636 East Alisal St, Salinias, CA, 93905, USA; 25 Evolutionary Systems Biology, SRI International, Room AE207, 333 Ravenswood Ave, Menlo Park, CA 94025, USA

## Abstract

**Background:**

*Pythium ultimum *is a ubiquitous oomycete plant pathogen responsible for a variety of diseases on a broad range of crop and ornamental species.

**Results:**

The *P. ultimum *genome (42.8 Mb) encodes 15,290 genes and has extensive sequence similarity and synteny with related *Phytophthora *species, including the potato blight pathogen *Phytophthora infestans*. Whole transcriptome sequencing revealed expression of 86% of genes, with detectable differential expression of suites of genes under abiotic stress and in the presence of a host. The predicted proteome includes a large repertoire of proteins involved in plant pathogen interactions, although, surprisingly, the *P. ultimum *genome does not encode any classical RXLR effectors and relatively few Crinkler genes in comparison to related phytopathogenic oomycetes. A lower number of enzymes involved in carbohydrate metabolism were present compared to *Phytophthora *species, with the notable absence of cutinases, suggesting a significant difference in virulence mechanisms between *P. ultimum *and more host-specific oomycete species. Although we observed a high degree of orthology with *Phytophthora *genomes, there were novel features of the *P. ultimum *proteome, including an expansion of genes involved in proteolysis and genes unique to *Pythium*. We identified a small gene family of cadherins, proteins involved in cell adhesion, the first report of these in a genome outside the metazoans.

**Conclusions:**

Access to the *P. ultimum *genome has revealed not only core pathogenic mechanisms within the oomycetes but also lineage-specific genes associated with the alternative virulence and lifestyles found within the pythiaceous lineages compared to the Peronosporaceae.

## Background

*Pythium *is a member of the Oomycota (also referred to as oomycetes), which are part of the heterokont/chromist clade [1,2] within the 'Straminipila-Alveolata-Rhizaria' superkingdom [3]. Recent phylogenies based on multiple protein coding genes indicate that the oomycetes, together with the uniflagellate hyphochytrids and the flagellates *Pirsonia *and *Developayella*, form the sister clade to the diverse photosynthetic orders in the phylum Ochrophyta [2,4]. Therefore, the genomes of the closest relatives to *Pythium *outside of the oomycetes available to date would be those of the diatoms *Thalassiosira *[5] and *Phaeodactylum *[6], and the phaeophyte algae *Ectocarpus *[7].

*Pythium *is a cosmopolitan and biologically diverse genus. Most species are soil inhabitants, although some reside in saltwater estuaries and other aquatic environments. Most *Pythium *spp. are saprobes or facultative plant pathogens causing a wide variety of diseases, including damping-off and a range of field and post-harvest rots [8-12]. *Pythium *spp. are opportunistic plant pathogens that can cause severe damage whenever plants are stressed or at a vulnerable stage. Some species have been used as biological control agents for plant disease management whereas others can be parasites of animals, including humans [13-15]. The genus *Pythium*, as currently defined, contains over a hundred species, with most having some loci sequenced for phylogeny [16]. *Pythium *is placed in the Peronosporales *sensu lato*, which contains a large number of often diverse taxa in which two groups are commonly recognized, the paraphyletic Pythiaceae, which comprise the basal lineages of the second group, the Peronosporaceae.

The main morphological feature that separates *Pythium *lineages from *Phytophthora *lineages is the process by which zoospores are produced from sporangia. In *Phytophthora*, zoospore differentiation happens directly within the sporangia, a derived character or apomorphism for *Phytophthora*. In *Pythium*, a vesicle is produced within which zoospore differentiation occurs [12]; this is considered the ancestral or plesiomorphic state. There is a much wider range of sporangial shapes in *Pythium *than is found in *Phytophthora *(see [17] for more detailed comparison). Biochemically, *Phytophthora *spp. have lost the ability to synthesize thiamine, which has been retained in *Pythium *and most other oomycetes. On the other hand, elicitin-like proteins are abundant in *Phytophthora *but in *Pythium *they have been mainly found in the species most closely related to *Phytophthora *[18-20]. Many *Phytophthora *spp. have a rather narrow plant species host range whereas there is little host specificity in plant pathogenic *Pythium *species apart from some preference shown for either monocot or dicot hosts. Gene-for-gene interactions and the associated cultivar/race differential responses have been described for many *Phytophthora *and downy mildew species with narrow host ranges. In constrast, such gene-for-gene interactions or cultivar/race differentials have never been observed in *Pythium*, although single dominant genes were associated with resistance in maize and soybean against *Pythium inflatum *and *Pythium aphanidermatum*, respectively [21,22], and in common bean against *P. ultimum *var. *ultimum *(G Mahuku, personal communication). Lastly, in the necrotroph to biotroph spectrum, some *Pythium *spp. are necrotrophs whereas others behave as hemibiotrophs like *Phytophthora *spp. [23].

*P. ultimum *is a ubiquitous plant pathogen and one of the most pathogenic *Pythium *spp. on crop species [13]. It does not require another mating type for sexual reproduction as it is self-fertile - that is, homothallic - but outcrossing has been reported [24]. *P. ultimum *is separated into two varieties: *P. ultimum *var. *ultimum *is the most common and pathogenic group and produces oospores but very rarely sporangia and zoospores, whereas *P. ultimum *var. *sporangiiferum *is a rare and less pathogenic group that produces both oospores and sporangia [12]. The isolate (DAOM BR144 = CBS 805.95 = ATCC 200006) reported in this study belongs to *P. ultimum *var. *ultimum *and was found to be the most representative strain [16,25,26]. We use *P. ultimum *to refer to *P. ultimum *var. *ultimum *unless stated otherwise.

In this study, we report on the generation and analysis of the full genome sequence of *P. ultimum *DAOM BR144, an isolate obtained from tobacco. The genomes of several plant pathogenic oomycetes have been sequenced, including three species of *Phytophthora *(*Ph. infestans*, *Ph. sojae*, and *Ph. ramorum *[27,28]), allowing the identification and improved understanding of pathogenicity mechanisms of these pathogens, especially with respect to the repertoire of effector molecules that govern the outcome of the plant-pathogen interaction [27-30]. To initially assess the gene complement of *P. ultimum*, we generated a set of ESTs using conventional Sanger sequencing coupled with 454 pyrosequencing of *P. ultimum *(DAOM BR144) hyphae grown in rich and nutrient-starved conditions [31]. These transcriptome sequence data were highly informative and showed that *P. ultimum *shared a large percentage of its proteome with related *Phytophthora *spp. In this study, we report on the sequencing, assembly, and annotation of the *P. ultimum *DAOM BR144 genome. To gain insight into gene function, we performed whole transcriptome sequencing under eight growth conditions, including a range of abiotic stresses and in the presence of a host. While the *P. ultimum *genome has similarities to related oomycete plant pathogens, its complement of metabolic and effector proteins is tailored to its pathogenic lifestyle as a necrotroph.

## Results and discussion

### Sequence determination and gene assignment

Using a hybrid strategy that coupled deep Sanger sequencing of variable insert libraries with pyrosequencing, we generated a high quality draft sequence of the oomycete pathogen *P. ultimum *(DAOM BR144 = CBS 805.95 = ATCC 200006). With an N_50 _contig length of 124 kb (1,747 total) and an N_50 _scaffold length of 773,464 bp (975 total), the *P. ultimum *assembly represents 42.8 Mb of assembled sequence. Additional metrics on the genome are available in Additional file 1.

*P. ultimum*, *Ph. sojae *and *Ph. ramorum *differ in mating behaviour: *P. ultimum and Ph. sojae *are homothallic while *Ph. ramorum *is heterothallic. The outcrossing preference in *Ph. ramorum *is reflected in the 13,643 single nucleotide polymorphisms identified in this species versus 499 found in the inbreeding *Ph. sojae *[27]. Although the *Ph. sojae *genome size is twice that of *P. ultimum*, a large number (11,916) of variable bases (that is, high quality reads in conflict with the consensus) were present within the DAOM BR144 assembly, indicating that the *in vitro *outcrossing reported for *P. ultimum *[24] might be common in nature.

The final genome annotation set (v4) contained 15,297 genes encoding 15,329 transcripts (15,323 protein coding and 6 rRNA coding) due to detection of alternative splice forms. Global analysis of the intron/exon structure revealed that while there are examples of intron-rich genes in the *P. ultimum *genome, the majority of genes tend to have few introns, with an average 1.6 introns occurring per gene that are relatively short (average intron length 115 bp), consistent with that of *Ph. infestans *(1.7 introns per gene, 124 bp average intron length). Coding exons in the *P. ultimum *genome tend to be relatively long when compared to other eukaryotes [32-40], having an average length of 498 bp, with 38.9% of the *P. ultimum *genes encoded by a single exon. This is comparable to that observed in *P. infestans*, in which the average exon is 456 bp with 33.1% encoding single exon genes.

In eukaryotic genomes such as that of *Arabidopsis thaliana *and human, 79% and 77% of all genes contain an InterPro domain, respectively. In comparison, only 60% of all *P. ultimum *genes contain an InterPro protein domain, which is comparable to that observed with *Phytophthora *spp. (55 to 66%). This is most likely attributable to the higher quality annotation of the human and *Arabidopsis *proteomes and, potentially, the lack of representation of oomycetes in protein databases.

Earlier transcriptome work with strain DAOM BR144 involved Sanger and 454 pyrosequencing of a normalized cDNA library constructed from two *in vitro *growth conditions [31]. When mapped to the DAOM BR144 genome, these ESTs (6,903 Sanger- and 21,863 454-assembled contigs) aligned with 10,784 gene models, providing expression support for 70.5% of the gene set. To further probe the *P. ultimum *transcriptome and to aid in functional annotation, we employed mRNA-Seq [41] to generate short transcript reads from eight growth/treatment conditions. A total of 71 million reads (2.7 Gb) were mapped to the DAOM BR144 genome and 11,685 of the 15,297 loci (76%) were expressed based on RNA-Seq data. Collectively, from the Sanger, 454, and Illumina transcriptome sequencing in which eight growth conditions, including host infection, were assayed, transcript support was detected for 13,103 genes of the 15,291 protein coding genes (85.7%). When protein sequence similarity to other annotated proteins is coupled with all available transcript support, only 190 of the 15,291 protein coding genes lack either transcript support or protein sequence similarity (Table S1 in Additional file 2).

#### Repeat content in DAOM BR144

In total, 12,815 repeat elements were identified in the genome (Table S2 in Additional file 2). In general, the relatively low repeat content of the *P. ultimum *genome (approximately 7% by length) is similar to what would be expected for small, rapidly reproducing eukaryotic organisms [42,43]. While the repeat content is much lower than that of the oomycete *Ph. infestans *[28], the difference is likely due to the presence of DNA methylases identified by protein domain analyses in the *P. ultimum *genome, which have been shown to inhibit repeat expansion [44]. Interestingly, the oomycete *Ph. infestans *lacks DNA methylase genes, the absence of which is believed to contribute to repeat element expansion within that organism, with repeats making up > 50% of the genome [27,28,45].

#### Mitochondrial genome

The *P. ultimum *DAOM BR144 mitochondrial genome is 59,689 bp and contains a large inverted repeat (21,950 bp) that is separated by small (2,711 bp) and large (13,078 bp) unique regions (Figure S1 in Additional file 3). The *P. ultimum *DAOM BR144 mitochondrion encodes the same suite of protein coding (35), rRNA (2), and tRNA (encoding 19 amino acids) genes present in other oomycetes such as *Phytophthora *and *Saprolegnia *[46-48]. However, the number of copies is different due to the large inverted repeat as well as some putative ORFs that are unique to *P. ultimum *(Additional file 1). No insertions of the mitochondrial genome into the nuclear genome were identified.

### Proteins involved in plant-pathogen interactions

Comparative genome analyses can reveal important differences between *P. ultimum *and the Peronosporaceae that may contribute to their respective lifestyles, that is, the non-host specific *P. ultimum *and the host specific *Phytophthora *spp. We utilized two approaches to probe the nature of gene complements within these two clades of oomycetes. First, using the generalized approach of examining PANTHER protein families [49], we identified major lineage-specific expansions of gene families. Second, through targeted analysis of subsets of the *P. ultimum *proteome, including the secretome, effectors, proteins involved in carbohydrate metabolism, and pathogen/microbial-associated molecular patterns (PAMPs or MAMPs; for review see [50]), we revealed commonalities, as well as significant distinct features, of *P. ultimum *in comparison to *Phytophthora *spp.

#### Over-represented gene families

Several families involved in proteolysis were over-represented in *P. ultimum *compared to *Phytophthora *spp. (Table 1). This is primarily due to a massive expansion of subtilisin-related proteases (PTHR10795) in *P. ultimum *following the divergence from ancestors of *Phytophthora*. With regard to the total complement of serine proteases, the subtilisin family expansion in *P. ultimum *is somewhat counterbalanced by the trypsin-related serine protease family, which has undergone more gene duplication events in the *Phytophthora *lineage than the *Pythium *lineage. The metalloprotease M12 (neprolysin-related) family has also undergone multiple expansions, from one copy in the stramenopile most recent common ancestor, to three in the oomycete most recent common ancestor (and extant *Phytophthora*), then up to 12 in *P. ultimum *(data not shown).

**Table 1:** Major lineage-specific gene family expansions leading to differences in the P. ultimum gene complement compared to *Phytophthora*

Biological process	Comparison to *Phytophthora*	Protein family expansions (number of genes in *P. ultimum*/*Ph. ramorum*)
Proteolysis	Over-represented	HECT E3 ubiquitin ligase (56/28)
		Subtilisin-related serine protease S8A (43/7)
		Trypsin-related serine protease S1A (17/31)
		Pepsin-related aspartyl protease A1 (25/15)
		Metalloprotease M12 (12/3)
Intracellular	Under-represented	PTHR23257 S/T protein kinase (78/158)
signaling cascade		PTHR22985 S/T protein kinase (23/51)
		PTHR22982, CaM kinase (50/85)
		Phospholipase D (9/18)
Sulfur metabolism	Under-represented	Sulfatase (7/14)
		Cysteine desulfurylase (4/11)
		Sulfate transporter (10/18)
Water transport	Under-represented	Aquaporin (11/35)

E3 ligases are responsible for substrate specificity of ubiquitination and subsequent proteolysis, and secreted E3 ligases have been shown to act as effectors for pathogens by targeting host response proteins for degradation [51,52]. The HECT E3 family of ubiquitin-protein ligases (PTHR11254) apparently underwent at least two major expansions, one in the oomycete lineage after the divergence from diatoms and another in the *P. ultimum *lineage (Figure S2 in Additional file 3; Table 1). Most of the expansion in the *P. ultimum *lineage appears to be derived from repeated duplication of only two genes that were present in the *Pythium*-*Phytophthora *common ancestor. This expanded subfamily is apparently orthologous to the *UPL1 *and *UPL2 *genes from *A. thaliana*. Of the 56 predicted HECT E3 ligases in the *P. ultimum *genome (that had long enough sequences for phylogenetic analysis), 16 are predicted by SignalP [53] to have *bona fide *signal peptides, and another 10 have predicted signal anchors, a substantially larger number than reported for other oomycete genomes [54].

#### Under-represented gene families

Several gene families are significantly under-represented in the *P. ultimum *genome compared to *Phytophthora *(Table 1) and it appears that these are mostly due to expansions in the *Phytophthora *lineage rather than losses in the *Pythium *lineage, though the relatively long distance to the diatom outgroup makes this somewhat uncertain. These include the aquaporin family (PTHR19139), the phospholipase D family (PTHR18896; Additional file 1), four families/subfamilies of intracellular serine-threonine protein kinases, and three families involved in sulfur metabolism (sulfatases (PTHR10342), cysteine desulfurylases (PTHR11601) and sulfate transporters (PTHR11814)).

#### The *P. ultimum *secretome

As oomycete plant pathogens secrete a variety of proteins to manipulate plant processes [30,55], we predicted and characterized in detail the soluble secreted proteins of *P. ultimum*. The secretome of *P. ultimum *was identified by predicting secreted proteins using the PexFinder algorithm [56] in conjunction with the TribeMCL protein family clustering algorithm. The *P. ultimum *secretome is composed of 747 proteins (4.9% of the proteome) that can be clustered into 195 families (each family contains at least 2 sequences) and 127 singletons (Table S3 in Additional file 2; selected families are shown in Figure S3 in Additional file 3). Of these, two families and one singleton encode transposable-element-related proteins that were missed in the repeat masking process. The largest family contains 77 members, mostly ankyrin repeat containing proteins, of which only 3 were predicted to have a signal peptide. Notable families of secreted proteins include protease inhibitors (serine and cysteine), NPP1-like proteins (toxins), cellulose-binding elicitor lectin (CBEL)-like proteins with carbohydrate binding domains, elicitins and elicitin-like proteins, secreted E3 ubiquitin ligases (candidate effectors), cell-wall degrading enzymes, lipases, phospholipases, potential adhesion proteins, highly expanded families of proteases and cytochrome P450 (Table 2), and several families of 'unknown' function. A subset (88 proteins) of the secretome showed exclusive similarity to fungal sequences yet are absent in other eukaryotes (Table S4 in Additional file 2; see Table S1 in [57] for a list of organisms). These may represent shared pathogenicity proteins for filamentous plant pathogens, such as peroxidases (Family 68), CBEL-like proteins (Family 8), and various cell wall degrading enzymes and other hydrolases.

**Table 2:** Protein families implicated in plant pathogenesis: *P. ultimum *versus *Phytophthora *spp. or diatoms

	*P. ultimum*	*Ph. infestans*	*Ph. sojae*	*Ph. ramorum*	*Thalassiosira pseudonana *(diatom)	*Phaeodactylum tricornutum *(diatom)
ABC transporters^a^	140	137	141	135	57	65
Aspartyl protease families A1, A8^b^	29	16	16	18	ND	8
Crinklers (CRN-family)^a^	26	196	100	19	0	0
Cutinase^c^	0	4	13	4	0	ND
Cysteine protease families C1, C2, C56^a^	42	38	33	42	ND	11
Cytochrome P450s^b^	41	28	31	31	ND	10
Elicitin-like proteins^d^	24	40	57	50	0	0
Glycoside hydrolases^c^	180	277	301	258	59	ND
Lipases^d^	31	19	27	17	22	17
NPP1-like proteins (necrosis-inducing proteins)^d^	7	27	39	59	0	0
PcF/SCR-like^d^	3	16	8	1	0	0
Pectin esterases^c^	0	13	19	11	0	ND
Polysaccharide lyases^c^	29	67	54	49	0	ND
Phospholipases^d^	20	36	31	28	18	11
Protease inhibitors, all^d^	43	38	26	18	11	5
RXLR effectors^a^	0	563	350	350	0	0
Serine protease families S1A, S8, S10^b^	85	60	63	57	ND	31

^a^Data from manual curation/analyses. ^b^Data from PANTHER family analyses (MEROPS classification). ^c^Data from CAZy. ^d^Data from analysis of TRIBEMCL families. ND, not determined.

#### RXLR effectors

**Figure 1:**
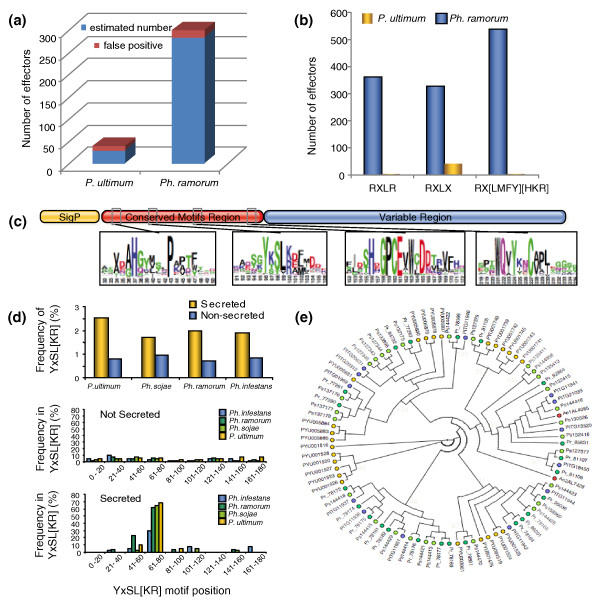
**An original repertoire of candidate effector proteins in *P. ultimum***. **(a) **The number of candidate RXLR effectors estimated by Hidden Markov Model (HMM) searches of predicted proteins with amino-terminal signal peptides. The numbers of false positives were derived from HMM searches of the permutated protein sequences. **(b) **The number of candidate RXLR effectors discovered by motif searching. The search was performed on the total set of six-frame translated ORFs from the genome sequences that encode proteins with an amino-terminal signal peptide. The motif RXLR and two more degenerate motifs, RXLX or RX[LMIFY][HKR], were required to occur within 100 amino acids of the amino termini. **(c) **The typical architecture of a YxSL[RK] effector candidate inferred from 91 sequences retrieved from *P. ultimum*, three *Phytophthora *genomes and *A. euteiches*. **(d) **The YxSL[RK] motif is enriched and positionally constrained in secreted proteins in *P. ultimum *and *Phytophthora *spp. The top graph compares the abundance of YxSL[RK]-containing proteins among secreted and non-secreted proteins from four oomycete genomes. The middle and bottom graphs show the frequency of the YxSL[RK] motif among non-secreted and secreted proteins, respectively, according to its position in the protein sequence. **(e) **Cladogram based on the conserved motifs region of the 91YxSL[KR] proteins, showing boostrap support for the main branches.

Many plant pathogens, especially biotrophic and hemi-biotrophic ones, produce effector proteins that either enter into host cells or are predicted to do so [27,58,59]. The genomes of *Ph. sojae*, *Ph. ramorum *and *Ph. infestans *encode large numbers (370 to 550) of potential effector proteins that contain an amino-terminal cell-entry domain with the motifs RXLR and dEER [28,29], which mediate entry of these proteins into host cells in the absence of pathogen-encoded machinery [60,61]. RXLR-dEER effectors are thought, and in a few cases shown, to suppress host defense responses, but a subset of these effectors can be recognized by plant immune receptors resulting in programmed cell death and disease resistance. To search for RXLR effectors in the genome of *P. ultimum*, we translated all six frames of the genome sequence to identify all possible small proteins, exclusive of splicing. Among these, a total of 7,128 translations were found to contain an amino-terminal signal peptide based on SignalP prediction. We then used the RXLR-dEER Hidden Markov Model (HMM) [29] to search the translations for candidate effectors and, as a control, the same set of translations following permutation of their sequences downstream of the signal peptide (Figure 1a). Only 35 sequences with significant scores were found in the non-permuted set while an average of 5 were found in 100 different permuted sets. In comparison to the *Ph. ramorum *secretome, 300 hits were found without permutation. Examination of the 35 significant sequences revealed that most were members of a secreted proteinase family [62] in which the RXLR motif was part of a conserved subtilisin-like serine protease domain of 300 amino acids in length, and thus unlikely to be acting as a cell entry motif. A string search was then performed for the RXLR motif within the amino terminus of each translation, 30 to 150 residues from the signal peptide. In this case, the number of hits was not significantly different between the real sequences and the permuted sequences. The same result was obtained with the strings RXLX and RX[LMFY][HKR] (Figure 1b). HMMs have been defined to identify carboxy-terminal motifs conserved in about 60% of RXLR-dEER effectors [29,63]. Searching the secretome and the permutated secretome with this HMM also identified no significant numbers of candidate effectors (data not shown). Blast searches with the most conserved *Phytophthora *effectors likewise produced no hits.

Based on synteny analysis of surrounding genes, a small number of *Phytophthora *effectors share conserved genomic positions [27]. Synteny analysis (see below) was used to identify the corresponding positions in the *P. ultimum *genome, but no predicted secreted proteins were found in those positions in the *P. ultimum *genome. A paucity of predicted RXLR effector sequences was reported previously in the transcriptome of *P. ultimum *[31]; the one candidate noted in the transcriptome sequence dataset has proven to be a false positive, matching the negative strand of a conserved transporter gene in the genome sequence. Therefore, we conclude that the *P. ultimum *genome lacks RXLR effectors that are abundant in other oomycetes, although this analysis does not rule out the possible presence of other kinds of effectors (see below). Nonetheless, the lack of RXLR effectors in *P. ultimum *is consistent with the absence of gene-for-gene interactions, all known instances of which in *Phytophthora *spp. involve RXLR effectors with avirulence activities.

#### CRN protein repertoire

In *Phytophthora *spp. the *Crinkler *(*crn*) gene family encodes a large class of secreted proteins that share a conserved amino-terminal LFLAK domain, which has been suggested to mediate host translocation and is followed by a major recombination site that forms the junction between the conserved amino terminus and diverse carboxy-terminal effector domains [28]. In sharp contrast to the RXLR effectors, the CRN protein family appears conserved in all plant pathogenic oomycete genomes sequenced to date. BLASTP searches of 16 well-defined amino-terminal domains from *Ph. infestans *against the *P. ultimum *predicted proteome identified 18 predicted proteins within *P. ultimum *(BLAST cutoff of 1 × 10^-10^; Table S5 in Additional file 2). Examination of protein alignments revealed considerable conservation of the *P. ultimum *LFLAK domain. We used *P. ultimum *CRN sequence alignments to build an HMM and through HMM searches identified two additional predicted proteins with putative LFLAK-like domains. We assessed the distribution of candidate CRN proteins within *P. ultimum *families and identified six additional candidates in Family 64. Further examination of candidates confirmed the presence of LFLAK-like domains (Table S5 in Additional file 2). Surprisingly, only 2 (approximately 7.5%) of the 26 predicted CRN proteins were annotated as having signal peptides (Table S5 in Additional file 2). Two additional CRNs (PYU1_T003336 and PYU1_T002270) have SignalP v2.0 HMM scores of 0.89 and 0.76, respectively, which although below our stringent cutoff of 0.9 may still suggest potential signal peptides. Several of the remaining genes have incomplete ORFs and gene models, suggesting a high frequency of CRN pseudogenes as previously noted in *Ph. infestans *[28]. All 26 amino-terminal regions were aligned to generate a sequence logo. These analyses revealed a conserved LxLYLAR/K motif that is shared amongst *P. ultimum *CRN proteins (Figure S4 in Additional file 3) and is followed by a conserved WL motif. The LxLYLAR/K motif is closely related to the F/LxLYLALK motif found in *Aphanomyces euteiches *[64]. Consistent with results obtained in other oomycete genomes, we found that the LxLYLAR/K motif was located between 46 and 64 amino acids after the methionine, followed by a variable domain that ended with a conserved motif at the proposed recombination site (HVLVxxP), reflecting the modular design of CRN proteins in the oomycetes (Figure S4 in Additional file 3). This recombination site, which is characteristic for the DWL domain, was found highly conserved in 11 of the putative *P. ultimum CRN *genes, consisting of an aliphatic amino acid followed by a conserved histidine, another three aliphatic amino acids, two variable amino acids and a conserved proline. In a phylogenetic analysis, these 11 genes were predominantly placed basal to the validated CRNs from *Phytophthora *(Figure S5 in Additional file 3). Although the CRN-like genes in *Pythium *are more divergent than the validated CRNs of *Phytophthora *(Figure S5 in Additional file 3), both the recombination site and the LxLYLAR/K-motif, which is a modification of the prominent LxLFLAK-motif present in most *Phytophthora *CRNs, show a significant degree of conservation, highlighting that the CRN family, greatly expanded in *Phytophthora *[28], had already evolved in the last common ancestor of *P. ultimum *and *Phytophthora*.

#### A novel family of candidate effectors

In the absence of obvious proteins with an amino-terminal RXLR motif, we used other known features of effectors to identify candidate effector families in *P. ultimum*. *Ph. infestans *RXLR effectors are not only characterized by a conserved amino-terminal translocation domain but also by their occurrence in gene-sparse regions that are enriched in repetitive DNA [28]. Based on the length of the flanking non-coding regions, the distribution of *P. ultimum *genes is not multimodal as was observed in *Ph. infestans *(Figure S6 in Additional file 3). However, relative to the rest of the genes, *P. ultimum *secretome genes more frequently have long flanking non-coding regions (Figure S7 in Additional file 3). In addition, the secretome genes show a higher proportion of closely related paralogs, suggesting recent duplications in *P. ultimum *(Figure S7 in Additional file 3) and indicating that the secretome genes may have distinct genome organization and evolution as noted in *Phytophthora *spp. [28,57]. Using genome organization properties to identify families of secreted proteins in *P. ultimum *that could correspond to novel effector candidates, we sorted the 194 secretome families based on highest rate of gene duplication, longest flanking non-coding region, and lowest similarity to *Ph. infestans *proteins (see Figure S8 in Additional file 3 for examples). One relatively large family of secreted proteins, Family 3, stood out because it fulfilled the three criteria and included proteins of unknown function. BlastP similarity searches identified similar sequences only in oomycete species (*Phytophthora *spp. and *A. euteiches*). Furthermore, of the 44 family members in *P. ultimum *for which transcripts could be detected, 32 (73%) were induced more than 2-fold during *Arabidopsis *infection compared to mycelia, with 5 members induced more than 40-fold. In total, we identified a set of 91 predicted secreted proteins with similarity to Family 3 proteins from the various oomycete species (Additional file 4). Multiple alignments of these proteins, along with motif searches, identified a YxSL[RK] amino acid motif (Figure 1c). This motif is at least two-fold enriched in secreted proteins compared to non-secreted proteins in four oomycete species (Figure 1d). In addition, the YxSL[RK] motif is positionally constrained between positions 61 and 80 in secreted oomycete proteins only (Figure 1d). The 91 YxSL[RK] proteins show a modular organization with a conserved amino-terminal region, containing four conserved motifs, followed by a highly variable carboxy-terminal region (Figure 1c; Figure S9 in Additional file 3) as reported for other oomycete effectors [30]. Phylogenetic analyses of the YxSL[RK] family revealed four main clades and suggest an expansion of this family in *Phytophthora *spp. (Figure 1e).

The YxSL[RK] motif appears to be a signature for a novel family of secreted oomycete proteins that may function as effectors. It is intriguing that the YxSL[RK] motif shares some similarity in sequence and position with the canonical RXLR motif, a resemblance increased by the fact that the variable amino acid is a basic amino acid (lysine) in 28 out of the 91 family members. Whether the YxSL[RK] motif defines a host-translocation domain as noted for RXLR effectors remains to be determined.

#### Detection of *P. ultimum *by the host

Detection of pathogens through the perception of PAMPs/MAMPs leads to the induction of plant immune responses (for review, see [50]). Oomycetes produce various and specific molecules able to induce defense responses like elicitins (for review, see [65]), but only two oomycete cell-surface proteins containing a MAMP have been characterized: a transglutaminase [66] and a protein named CBEL [67]. Genes encoding both of these cell-surface proteins were detected in *P. ultimum *(Additional file 1), suggesting that *P. ultimum *produces typical oomycete MAMPs, which can be efficiently perceived by a wide range of plant species. The occurrence of PAMPs/MAMPs in *P. ultimum *suggests that this pathogen must have evolved mechanisms to evade PAMP-triggered immunity. This could occur through a necrotrophic mechanism of infection or using the candidate effector proteins described above.

#### Metabolism of complex carbohydrates

A total of 180 candidate glycoside hydrolases (GHs) were identified in *P. ultimum *using the CAZy annotation pipeline [68]. This number is apparently similar to those reported previously for *Ph. ramorum *(173), *Ph. sojae *(190), and *Ph. infestans *(157) [27,28]. However, when the CAZy annotation pipeline was applied to *Ph. sojae*, *Ph. ramorum *and *Ph. infestans*, 301, 258 and 277 GHs were found, respectively, nearly twice the number present in *P. ultimum *(Table 2). Among these we identified putative cellulases belonging to families GH5, GH6 and GH7. All six GH6 candidate cellulases harbor secretion signals. Only one GH6 protein contains a CBEL domain at the carboxyl terminus. Three contain a transmembrane domain and one contains a glycosylphosphatidylinisotol anchor, features suggesting that these proteins may be targeting the oomycete cell wall rather than plant cell walls. The *P. ultimum *strain studied here could not grow when cellulose was the sole carbon source (Table 3; Figure S10 in Additional file 3).

**Table 3:** Growth comparison of *P. ultimum *DAOM BR 144 on different carbon sources and the pH of the medium after 7 days

	DAOM BR144	
	
Carbon source	Mycelium density	pH on day 7
No carbon	-	5.1
25 mM D-glucose	+++	2.9
25 mM D-fructose	+++	2.9
25 mM D-xylose	-	5
25 mM L-arabinose	-	5
25 mM cellobiose	+++	4
25 mM sucrose	+++	3.2
1% cellulose	-	5.2
1% birch wood xylan	-	4.7
1% soluble starch	+++	3.5
1% citrus pectin*	+	5

The symbols indicate poor growth (+), moderate growth (++), good growth (+++), very good growth (++++), or growth less than or equal to the no-carbon medium (-). The data are the average of the two duplicates used for this experiment.

Cutinases are a particular set of esterases (CAZy family CE5) that cleave cutin, a polyester composed of hydroxy and hydroxyepoxy fatty acids that protects aerial plant organs. No candidate cutinases could be found in the *P. ultimum *genome. Cutinase activity was reported in culture filtrates of *P. ultimum*, but its growth was not supported on apple cutin [69] and low levels of fatty acid esterase were detected in *P. ultimum *only in 21-day-old culture [70]. The absence of recognizable cutinases suggests these enzymes are not critical for penetration and infection by *P. ultimum*, which attacks young, non-suberized roots and penetrates tissues indirectly through wounds. This contrasts with the number of putative cutinases identified in several *Phytophthora *spp. [27,71-73], which presumably promote penetration of leaf and stem tissues that are protected by a thick cuticle or colonization of heavily suberized root and bark tissue.

The xylan degrading capacity of *P. ultimum *appears to be limited, if not totally absent. No members of the GH10 and GH11 families encoding endoxylanases essential for xylan degradation could be found. Furthermore, families involved in the removal of xylan side chains or modifications such as GH67, CE3, and CE5 are absent while families CE1 and CE2 contain only a limited number of members. The lack of significant xylan digestion was confirmed by the absence of growth when xylan was used as a carbon source (Table 3; Figure S10 in Additional file 3), consistent with previous work on *P. ultimum *and other *Pythium *spp. [70].

Pectinases play a key role in infection by *Pythium *spp. [74]. Twenty-nine candidate pectin/pectate lyases (PL1, PL3 and PL4 families) are present in *P. ultimum *while the genomes of *Phytophthora *spp. [27,28] encode even larger PL families (Table 2). In *P. ultimum*, the set of pectin lyases is complemented by 11 pectin hydrolases from family GH28, several of which having been functionally characterized in various *Phytophthora *spp. [75-78]. *P. ultimum *lacks pectin methylesterases as well as genes encoding family GH88 and GH105 enzymes and therefore cannot fully saccharify the products of pectin/pectate lyases, consistent with previous reports of incomplete pectin degradation and little or no galacturonic acid production during *P. ultimum *infection of bentgrass [79]. The data from the carbon source utilization experiment (Table 3; Figure S10 in Additional file 3) show only limited growth on medium with citrus pectin as the sole carbon source.

We also observed that the *P. ultimum *genome encodes candidate GH13 α-amylases, GH15 glucoamylase and a GH32 invertase, suggesting that plant starch and sucrose are targeted. The growth data confirm these observations, with excellent growth on soluble starch and sucrose (Figure S10 in Additional file 3).

The CAZy database also contains enzymes involved in fungal cell wall synthesis and remodeling. Cell walls of oomycetes differ markedly from cell walls of Fungi and consist mainly of glucans containing β-1,3 and β-1,6 linkages and cellulose [80-82]. The *P. ultimum *genome encodes four cellulose synthases closely related to their orthologs described for *Ph. infestans *[82]. The genome also specifies a large number of enzyme activities that may be involved in the metabolism of β-1,3- and β-1,6-glucans (Additional file 1), as well as a large set of candidate β-1,3-glucan synthases likely involved in synthesis of cell wall β-glucans and in the metabolism of mycolaminaran, the main carbon storage compound in *Phytophthora *and *Pythium *spp. [81,83,84].

### Reponses to fungicide

Metalaxyl and its enantiopure R form mefenoxam have been used widely since the 1980s for the control of plant diseases caused by oomycetes [17,85]. The main mechanism of action of this fungicide is selective inhibition of ribosomal RNA synthesis by interfering with the activity of the RNA polymerase I complex [86]. *P. ultimum *DAOM BR144 is sensitive to mefenoxam at concentrations higher than 1 μl/l (data not shown) and 45 genes were expressed five-fold or more when *P. ultimum *was exposed to it (Table S6 in Additional file 2). Active ABC pump efflux systems are important factors for drug and antifungal resistance in Fungi and oomycetes [87-91]. Although the substrates transported by ABC proteins cannot be predicted on the basis of sequence homology, it is clear that these membrane transporters play a key role in the adaptation to environmental change. Three pleiotropic drug resistance proteins (ABC, subfamily G) were strongly up-regulated (> 27-fold) in response to mefenoxam. These genes arose from a tandem duplication event but remain so similar that it is possible that only one of these genes is actually up-regulated under these conditions due to our inability to uniquely map mRNA-seq reads when there are highly similar paralogs. A fourth gene and a member of the multidrug resistance associated family was also up-regulated more than nine-fold. Notably, the ABC transporters in *P. ultimum *that were up-regulated are distinct from those that were up-regulated in *Ph. infestans *in response to metalaxyl [92], indicating that a unique set of ABC transporters may be involved in the response to the fungicide in *P. ultimum*. Three genes coding for E3 ubiquitin-protein ligase were more than 18-fold up-regulated in response to mefenoxam compared to the control, but not in the other tested conditions. Ubiquitin/proteasome-mediated proteolysis is activated in response to stress - such as nutrient limitation, heat shock, and exposure to heavy metals - that may cause formation of damaged, denatured, or misfolded proteins [93,94]. Thus, increased expression of these enzymes in *P. ultimum *exposed to mefenoxam might be related to decreased synthesis of rRNA and expression of aberrant proteins.

### Comparative genomics

#### Zoospore production

*P. ultimum *does not typically exhibit release of zoospores from sporangia in culture [12] but zoospore release directly from aged oospores has been reported [95]. Comparative genomics with well studied whiplash flagellar proteins from the green algae *Chlamydomonas reinhardtii *and other model organisms indicates that indeed *P. ultimum *does have the necessary genetic complement for flagella. Orthologs of tinsel flagellar mastigoneme proteins have also been identified in *P. ultimum *through comparison to those studied in *Ochromonas danica*, a unicellular member of the Straminipila kingdom. Overall, approximately 100 putative whiplash and tinsel flagellum gene orthologs were identified in *P. ultimum *(Table S7 in Additional file 2) with corresponding orthologs present in *Ph. infestans*, *Ph. sojae*, and *Ph. ramorum*. Expression of flagellar orthologs was observed in 8 growth conditions used in whole transcriptome sequencing, although 14 putative flagellar orthologs for axonemal dynein and kinesin and intraflagellar transport did not show expression in any condition.

#### Cadherins, an animal gene family found in oomycetes

Perhaps the most remarkable discovery relative to gene family expansion is that there are four *P. ultimum *genes that encode cadherins. Previously, members of this gene family have only been found in metazoan genomes (and the one fully sequenced genome from the clade of nearest relatives, the choanoflagellate *Monosiga brevicollis*). Cadherins are cell adhesion proteins that presumably evolved at the base of the clade containing metazoans and choanoflagellates [96]. Cadherin-related proteins are encoded in several bacterial genomes, but these bacterial proteins lack important calcium ion-binding motifs (the LDRE and DxND motifs) found in the extracellular (EC) repeat domains of 'true' cadherins [97]. The cadherin genes in *P. ultimum *do contain these motifs, and this is therefore the first report of true cadherins in a genome outside the metazoans/choanoflagellates. In metazoans, but not in choanoflagellates, some cadherins also contain an intracellular catenin-binding domain (CBD) that connects intercellular binding via EC domains to intracellular responses such as cytoskeletal changes. A search of predicted gene models with the PANTHER HMMs for cadherins (PTHR10596) identified two genes containing cadherin EC domains in the *Ph. infestans *genome, but none in the *Ph. ramorum*, *Ph. sojae *and *Phaeodactylum tricornutum *genomes. The identification of cadherin EC domains in both *P. ultimum *and *Ph. infestans *led us to postulate that such genes may also exist in other *Phytophthora *genomes that were not found in the original analysis of these genomes. Indeed, a TBLASTN search of genomic DNA using the predicted *P. ultimum *cadherin domain-containing proteins identified one putative cadherin-containing ORF in the *Ph. sojae *genome and four in the *Ph. ramorum *genome. The *P. ultimum *cadherin genes contain between 2 and 17 full-length cadherin EC domains, as predicted by the Pfam database [98] at the recommended statistical significance threshold, and likely a number of additional cadherin domains that have been truncated and/or have diverged past this similarity threshold. The genes from the *Phytophthora *genomes each contain between one and seven intact cadherin EC domains, though we did not attempt to construct accurate gene models for the *Phytophthora *genes. None of the oomycete cadherins appear to have the catenin-binding domain, nor do these genomes appear to encode a β-catenin gene, so like in *M. brevicollis*, the β-catenin-initiated part of the classical metazoan cadherin pathway appears to be absent from oomycetes.

**Figure 2:**
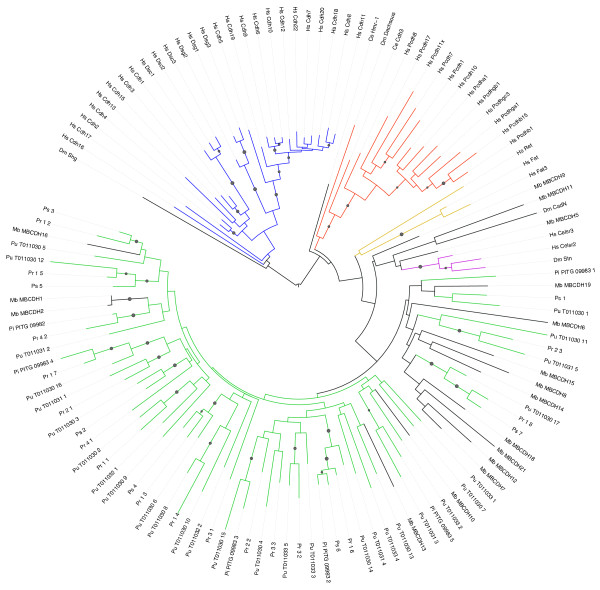
**Phylogenetic tree of the cadherin family, showing all members of the novel oomycete subfamily (green) and their relationships to representative metazoan and choanoflagellate cadherins**. The major clades of cadherins [96] are colored: C-1 (blue), Cr-1a and Cr-1b (red), C-2 (purple), and Cr-3 (orange). Most of the oomycete cadherins fall within a fairly distinct subfamily (green), though this subfamily has many long branches and also includes some cadherins from the choanoflagellate *M. brevicollis *(labeled starting with 'MB') that are also highly diverged from other cadherins. Reliable branches (bootstrap > 50%) are labeled with a circle. All full-length oomycete cadherin domains are shown, from *P. ultimum *(labeled starting with 'Pu' and ending with the number of the repeat relative to the amino terminus), *Ph. infestans *(labeled starting with 'Pi'), *Ph. sojae *(Ps) and *Ph. ramorum *(Pr). Other cadherins are from the human genome ('Hs') unless labeled starting with 'Dm' (*Drosophila melanogaster*) or 'Ce' (*Caenorhabditis elegans*). The figure was drawn using the iTOL tool [143].

In order to explore the evolution of these domains in the oomycetes, we performed a phylogenetic analysis. The first (amino-terminal) cadherin EC domain has been used to explore gene phylogeny among the cadherins [96,99], and to facilitate comparison we used both neighbor joining [100] and maximum likelihood (using the PhyML program [101,102]) to estimate a phylogenetic tree for these same sequences together with all of the intact cadherin domains from the *P. ultimum *and *Ph. infestans *genomes (Figure 2). To generate a high-quality protein sequence alignment for phylogeny estimation, we used the manual alignment of Nollet *et al*. [99] as a 'seed' for alignment of other sequences using MAFFT [102]. We found that all of the oomycete domains fall within a single clade. However, this clade is broad and also contains several cadherins from the choanoflagellate *M. brevicollis*, as well as some of the more divergent metazoan cadherins (Cr-2 and Cr-3 subfamilies). In general, the branches in this clade are very long, making phylogenetic reconstruction somewhat unreliable (all branches with bootstrap values > 50% are marked with a circle in Figure 2). Nevertheless, most of the cadherin domains found in *P. ultimum *are reliably orthologous to domains in one or more *Phytophthora *species, suggesting descent from a common ancestor by speciation. The most notable example is for the genes PITG_09983 and PYU1_T011030, in which a region spanning three consecutive EC repeats appears to have been inherited by both species from that common ancestor (apparently followed by substantial duplication and rearrangement of individual cadherin domains). These repeats are also apparently orthologous to repeats in both *Ph. sojae *and *Ph. ramorum*. The oomycete cadherins may have been initially obtained either vertically (by descent from the common ancestor with metazoans) or horizontally (by transfer of metazoan DNA long after divergence). No cadherins have been found in genomes sequenced from other clades more closely related to either oomycetes (for example, diatoms and alveolates) or the metazoan/choanoflagellates (for example, Fungi and amoebozoa). This means that, if cadherins were present in the most recent common ancestor of oomycetes and metazoans, these genes must have been lost independently in all of these other diverging lineages. Given the data currently available, it is more probable that at least one horizontal cadherin gene transfer event occurred from a choanoflagellate or metazoan to an oomycete ancestor, prior to the divergence of *Pythium *from *Phytophthora*. The source of the metazoan DNA may have been a host of the ancestral oomycete, or possibly introduced by a virus. Nevertheless, the subsequent preservation of cadherin domains in at least two lineages of oomycetes over a substantial period of time suggests that the genes are likely to perform an important function, which remains to be explored.

#### Synteny with other oomycete plant pathogens

**Figure 3:**
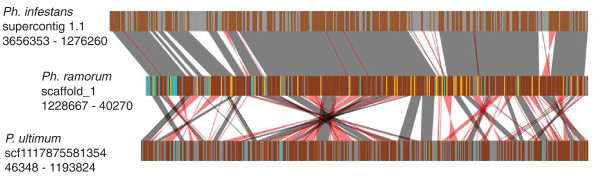
**Rearrangements in gene order in the *P. ultimum *genome relative to *Phytophthora *genomes**. Vertical brown bars indicate orthologs shared among *P. ultimum, Ph. infestans *and *Ph. ramorum*. Gold bars indicate orthologs shared only between *Ph. infestans *and *Ph. ramorum*. Turquoise bars indicate genes with orthologs in other regions of the compared genomes (that is, non-syntenic orthologs). Grey bars indicate genes without orthologs. Gray and red shaded connections indicate blocks of syntenic orthologs with the same or opposite relative transcriptional orientations, respectively. Non-coding regions of the genome are not depicted.

A phylogenetic approach (PHRINGE [103]) was used to identify *P. ultimum *proteins orthologous to proteins encoded in the genomes of *Ph. infestans*, *Ph. sojae*, and *Ph. ramorum*. Of the 15,322 proteins predicted from the *P. ultimum *genome sequence, 12,230 were identified as orthologous to a protein in at least one *Phytophthora *genome sequence. A total of 11,331 proteins were identified as orthologs common to all three *Phytophthora *spp., and of these, 8,504 had identifiable orthologs in *P. ultimum*. PHRINGE was also used to examine the conservation of gene order (synteny) between the *Phytophthora *and *P. ultimum *genomes. As previously described [27], the gene order of orthologs is very highly conserved among *Phytophthora *spp. In *P. ultimum *the ortholog content was very similar between broad regions of the *P. ultimum *and *Phytophthora *genomes, but the local gene order was greatly rearranged, primarily as a result of inversions. Only short runs of up to 10 orthologs were found to be collinear, whereas runs of more than 100 could be identified between the *Phytophthora *spp. Figure 3 shows an example spanning a well-assembled region of the *Ph. infestans*, *Ph. ramorum *and *P. ultimum *genome sequences. In *Ph. ramorum*, the region spans 1.18 Mb and 383 predicted genes and in *P. ultimum *the region spans 1.15 Mb and 435 predicted genes. Of these genes, 286 are identified as orthologs. In the *Ph. ramorum *sequence there are an additional 38 genes with orthologs in *Ph. infestans *but not in *P. ultimum*. Due to a much larger number of repeat sequences, and expanded gene numbers, the corresponding region in *Ph. infestans *spans 2.38 Mb and 499 predicted genes, but the order of the orthologous genes is highly conserved with that of *Ph. ramorum*. The *Ph. sojae *genome shows similar conservation of gene order in this region but for simplicity is not shown.

## Conclusions

Analysis of the *P. ultimum *genome sequence suggests that not all oomycete plant pathogens contain a similar 'toolkit' for survival and pathogenesis. Indeed, *P. ultimum *has a distinct effector repertoire compared to *Phytophthora *spp., including a lack of the hallmark RXLR effectors, a limited number of *Crinkler *genes, and a novel YxSL[RK] family of candidate effectors. The absence of any convincing RXLR effectors from the predicted proteome of *P. ultimum*, first noted by Cheung *et al*. [31] and rigorously confirmed here, provides a striking contrast to the *Phytophthora *genomes. RXLR effectors are also absent from the proteome of *A. euteiches*, a member of the Saprolegniales, which was predicted from an EST collection [64]. It is possible that RXLR effectors are confined to oomycete pathogens in the family Peronosporaceae, and represent an adaptation to facilitate biotrophy. The absence of RXLR effectors from *P. ultimum *(and possibly all other species of the genus) may be functionally associated with the very broad host range of *Pythium *pathogens. It also correlates with the lack of gene-for-gene resistance against *Pythium *and the fact that *Pythium *pathogens are generally restricted to necrotrophic infection of seedlings, stressed plants, and plant parts (for example, fruit) with diminished defenses against infection. In contrast to the RXLR effectors, the genome of *P. ultimum *does encode members of the Crinkler class of effectors, albeit not at the numbers present in *Phytophthora *genomes. These effectors may also enter host cells, and can trigger cell death [28]. They are also found in *A. euteiches *[64] and may represent a basal family of effectors that contribute to necrotrophy. This study uncovered a third family of secreted proteins conserved across all oomycetes sequenced so far with characteristics that suggest they might act inside host cells. These characteristics include high sequence variability, small size, hydrophilic nature, and a conserved RXLR-like motif (YxSL[KR]) with several family members specifically and highly expressed during infection. However, as yet no experimental data support this hypothesis.

**Figure 4:**
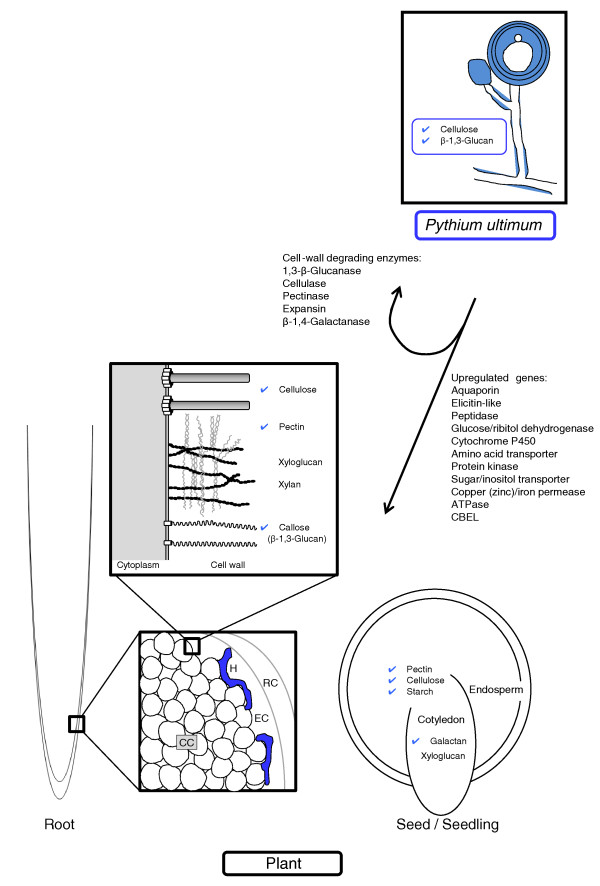
**The *P. ultimum *genome contains genes encoding enzyme activities necessary for the degradation of plant cell wall polysaccharides and storage sugars (blue ticks indicate the polysaccharides targeted by *P. ultimum *enzymes)**. Some activities are equally relevant for *P. ultimum*'s own cell wall metabolism. Degradation of the plant cell wall relies essentially on the action of cellulases and pectinases. Significantly, the absence of identified enzymes with xylanase, pectin methylesterase or cutinase activities is in agreement with previous studies of *P. ultimum *and other *Pythium *spp. [70,104,144]. For *Pythium*'s pathogenic action, penetration is primarily limited to wounded tissue, or to young roots and germinating seedlings with little or no suberized tissue. Penetration and root rot, for some *Pythium *spp., is limited to the first layers of cells (RC and EC) [104]. Other genes, including those coding for transporters, elicitin-like, and stress proteins, were upregulated when *P. ultimum *was grown in contact with *A. thaliana *seeds. CC, cortical cells; EC, epidermal cells; RC, root cap; H, hyphae. (Figure adapted from [104,144-146].)

The repertoire of metabolic genes within the *P. ultimum *genome reflects its pathogenic lifestyle (Figure 4). *P. ultimum *is an opportunistic pathogen of young seedlings and plant roots with little or no cuticle or heavily suberized tissue, consistent with lack of cutinase encoding genes. It is a poor competitor against secondary invaders of damaged plant tissues and soil organisms with better saprobic ability [13]. The *P. ultimum *genome contains a suite of GHs that fits well with an organism in this ecological niche. The genome encodes cellulases and pectinases that facilitate initial penetration and infection of the host, but it does not appear to use these plant polysaccharides as a major carbon source in culture and it lacks the ability to effectively degrade other complex polysaccharides such as xylan [70] (Table 3) and chitin [74,104]. As a primary pathogen that usually initiates infection, *P. ultimum *probably has first-hand access to easily degradable carbohydrates such as starch and sucrose. Following depletion of these carbon sources, it appears to focus on quick reproduction and production of survival structures [13] rather than switching its metabolism to the more difficult carbon sources such as plant cell wall polysaccharides. Intriguingly, the arsenal of *P. ultimum *enzymes targeting plant carbohydrates is strikingly similar to that found in the genome of the root-knot nematode *Meloidogyne incognita *[105], a root pathogen that also lacks xylanases yet has a strong pectin degrading capacity. In summary, access to the *P. ultimum *genome sequence has reinforced earlier hypotheses on pathogenesis and survival mechanisms in oomycete plant pathogens and has advanced our understanding of events at the plant-pathogen interface, especially during necrotrophy.

## Materials and methods

### Sequencing, assembly and autoclosure of *P. ultimum *DAOM BR144

*P. ultimum *(DAOM BR144 = CBS 805.95 = ATCC 200006) was sequenced using a whole-genome shotgun sequencing approach. Sequencing of three Sanger libraries generated 263,715 quality filtered reads (281,088 attempted reads). Three full runs of 454 FLX standard pyrosequencing [106] generated 1,296,941 reads. These were assembled by a 'shredding' pipeline [107] that generates pseudo-Sanger reads from the contigs of a Newbler assembly [106] of 454 reads and then assembles all of the reads with Celera Assembler [108]. This yielded 2,659 contigs in 714 scaffolds with an N_50 _contig size of 40,520 bp. The 1,945 intra-scaffold gaps were subjected to AutoClosure, an in-house pipeline that automates primer design, template re-array, and reaction orders. This produced 6,468 reads, of which 5,014 passed quality filtering. Subsequently, the Celera Assembler software was modified to accept 454 reads without shredding [109] and Celera Assembler 5.2 was run on the Sanger shotgun, 454 shotgun, and Sanger AutoClosure reads together. Contigs for the mitochondrial genome were identified and annotated separately with 16,277 sequences assembled for a greater than 200-fold coverage. The whole genome shotgun project has been deposited at NCBI [GenBank:ADOS00000000] along with the 454 reads [SRA:SRX020087], the mitochondrial genome [GenBank:GU138662], and the Sanger reads (NCBI Trace Archive under species code 'PYTHIUM ULTIMUM DAOM BR144'). The version described in this paper is the first version [WGS:ADOS01000000].

### Genome annotation

The *P. ultimum *genome annotations were created using the MAKER program [110]. The program was configured to use both spliced EST alignments as well as single exon ESTs greater than 250 bp in length as evidence for producing hint-based gene predictions. MAKER was also set to filter out gene models for short and partial gene predictions that produce proteins with fewer than 28 amino acids. The MAKER pipeline was set to produce *ab initio *gene predictions from both the repeat-masked and unmasked genomic sequence using SNAP [111], FGENESH [112], and GeneMark [113]. Hint-based gene predictions were derived from SNAP and FGENESH.

The EST sequences used in the annotation process were derived from Sanger and 454 sequenced *P. ultimum *DAOM BR144 ESTs [31] considered together with ESTs from dbEST [114] for *Aphanomyces cochlioides*, *Phytophthora brassicae*, *Phytophthora capsici*, *Phytophthora parasitica*, *Ph. sojae*, *Ph. infestans*, and *Pythium oligandrum*. Protein evidence was derived from the UniProt/Swiss-Prot protein database [115,116] and from predicted proteins for *Ph. infestans *[28], *Ph. ramorum *[27], and *Ph. sojae *[27]. Repetitive elements were identified within the MAKER pipeline using the Repbase repeat library [117] and RepeatMasker [45] in conjunction with a MAKER internal transposable element database [118] and a *P. ultimum *specific repeat library prepared for this work (created using PILER [119] with settings suggested in the PILER documentation). *Ab initio *gene predictions and hint-based gene predictions [110] were produced within the MAKER pipeline using FGENESH trained for *Ph. infestans*, GeneMark trained for *P. ultimum *via internal self-training, and SNAP trained for *P. ultimum *from a conserved gene set identified by CEGMA [110].

Following the initial MAKER run, a total of 14,967 genes encoding 14,999 transcripts were identified, each of which were supported by homology to a known protein or had at least one splice site confirmed by EST evidence. Additional *ab initio *gene predictions not overlapping a MAKER annotation were scanned for protein domains using InterProScan [120-122]. This process identified an additional 323 gene predictions; these were added to the annotation set, producing a total of 15,290 genes encoding 15,322 transcripts (referred to as v3). Selected genes within the MAKER produced gene annotation set were manually annotated using the annotation-editing tool Apollo [123]. The final annotation set (v4) contained 15,297 genes encoding 15,329 transcripts, including six rRNA transcripts.

Putative functions were assigned to each predicted *P. ultimum *protein using BLASTP [124] to identify the best homologs from the UniProt/Swiss-Prot protein database and/or through manual curation. Additional functional annotations include molecular weight and isoelectric point (pI) calculated using the pepstats program from the EMBOSS package [125], subcellular localization predicted with TargetP using the non-plant network [126], prediction of transmembrance helices via TMHMM [127], and PFAM (v23.0) families using HMMER [128] in which only hits above the trusted cutoff were retained. Expert annotation of carbohydrate-related enzymes was performed using the Carbohydrate-Active Enzyme database (CAZy) annotation pipeline [68].

### Transcriptome sequencing

Eight cDNA libraries were constructed to assess the expression profile of *P. ultimum*. Initially, plugs of 10% V8 agar containing *P. ultimum *strain DAOM BR144 were incubated for 1 day in yeast extract broth (YEB; 30 g/l sucrose, 1 g/l KH_2_PO_4_, 0.5 g/l MgSO_4_·7H_2_O, 0.5 g/l KCl, 10 mg/l FeSO_4_·7H_2_O, 1 g/l yeast extract) at 25°C with shaking (200 rpm). Approximately 50 mg of hyphae growing out of the plugs were then transferred to flasks containing media for the various expression assays. Mycelium was grown under the following conditions: 1, nutrient-rich YEB medium for 3 days at 25°C with shaking (200 rpm) and nutrient-starved Plich medium (S Kamoun, unpublished) for 10 days at 25°C in standing culture, as previously described [31]; 2, YEB medium under hypoxic conditions (oxygen concentration of 0.2%) for 1 and 3 days in standing liquid culture at 25°C; 3, YEB medium for 2 days at 25°C with shaking (200 rpm) followed by the addition of 1 and 100 μl/l of the fungicide mefenoxam (Subdue MAXX™, Novartis Crop Production, Greensboro, NC, USA) and subsequent incubation for an additional 0.25, 3 and 6 hours at the same temperature and with agitation; 4, YEB medium for the same time periods but without the addition of mefenoxam was included (mefenoxam control); 5, YEB medium for 2 days at 25°C with shaking (200 rpm) followed by a cold stress of 0°C with shaking (200 rpm) for 0.25, 3 and 6 hours; or 6, YEB medium for 2 days at 25°C with shaking (200 rpm) followed by exposure to heat stress of 35°C for 0.25, 3 and 6 hours; 7, YEB medium for 2 days at 25°C followed by exposure to 25°C for 0.25, 3 and 6 hours (temperature control); 8, 0.1% V8-juice medium containing surface-sterilized *A. thaliana *ecotype Columbia Col-0 seeds. Approximately 200 seeds were placed in the liquid medium at 25°C with shaking (200 rpm) for 1, 2 and 7 days. Mycelium of *P. ultimum *was then added and allowed to grow in contact with the seeds for 3 days.

For each condition listed above, mycelium was harvested, macerated in liquid nitrogen and RNA was extracted using TRIzol reagent (Invitrogen, Carlsbad, CA, USA) as described [31]. RNA was treated with DNAse (Promega RQ1 RNase-Free DNase, Madison, WI, USA) and 10 μg RNA was used to construct cDNA using the mRNA-Seq Sample Prep Kit (Illumina, San Diego, CA, USA), which was sequenced with Illumina Genome Analyzer (GA) II using version 3 sequencing reagents for 41 cycles. Base calling was carried out using the Illumina GA pipeline v1.4.

For each library, the filtered reads from the Illumina GA II pipeline were mapped using Tophat, a splice-site-aware short read mapper that works in conjunction with Bowtie short read aligner [129]. Reads were deposited in the NCBI Short Read Archive [SRA:SRP002690]. The minimum and maximum intron sizes were 5 bp and 15 kbp, respectively, for each Tophat run. The final annotation GFF3 file was provided to Tophat and expression values were calculated using reads per kilobase of exon model per million mapped reads (RPKM) [130]. The minimum RPKM for all eight conditions was 0, the median RPKM ranged from 5 to 8, while the maximum RPKM ranged from 10,182 in the YEP+Plich library to 32,041 from the 35°C temperature treatment. Using a RPKM value of 2.5 (approximately half of the median RPKM of all genes in each library) as a cutoff for expression, loci with differential expression in treatment versus control were identified (comparisons: hypoxia versus YEB+Plich; *Arabidopsis *versus YEB+Plich; mefenoxam treatment versus mefenoxam control; heat treatment versus temperature control; cold treatment versus temperature control; Table S6 in Additional file 2). The fold changes were calculated for loci with RPKM ≥ 2.5 in both treatment and control samples. Loci with control values < 2.5 RPKM but with expression in treatment conditions were flagged as 'U' as a true ratio could not be calculated. Loci with treatment values < 2.5 RPKM but with expression in control conditions were flagged as 'D' as a true ratio could not be calculated. Loci with RPKM < 2.5 in both treatment and control samples were flagged as 'N'.

### Identification of secreted proteins and effector families

The secretome of *P. ultimum *was identified using SignalP V2.0 program following the PexFinder algorithm as described previously [56]. In addition, sequences that were predicted to contain transmembrane domains or organelle targeting signals were omitted from the secretome. Each sequence in the secretome was searched against two 'Darwin' databases [57] that were compiled from > 50 eukaryote whole proteomes from major phylogenetic branches using BLASTP with an E-value cutoff of 1 × 10^-3^. One database contained sequences only from Fungi and the other contained the sequences from other organisms excluding the Fungi and oomycetes. Protein sequences of the secretome were clustered into families along with their related non-secretory proteins by using the TRIBE-MCL algorithm [131] using BLASTP with an E-value cutoff of 1 × 10^-10^. Each family was named according to the existing annotation of the member sequences. Families and singletons were searched against Pfam-A release 24.0 using the HMMER3 beta 3 hmmsearch with trusted cutoffs to detect any transposable-element-related proteins that may have been missed in the repeat masking process. Families or singletons where at least 50% of the members matched transposon-associated Pfam domains were manually curated to identify and exclude true transposon-related sequences from the secretome.

For the analysis of genome organization, *P. ultimum *predicted genes were binned according to the length of their flanking non-coding regions (FIRs). FIRs were computed using predicted gene coordinates on scaffolds. Binning according to 5' FIRs and 3' FIRs was performed along the x-aixs and y-axis, respectively, using conditional counting functions. Logarithmic size was chosen for the bins in order to allow a maximum dispersion of the values. A color code was used to represent the number of genes or average values in bins. Average values were computed for bins containing a minimum of three genes.

Motif searches were done using the MEME [132] prediction server with default parameters except the following: min width = 4; max width = 12; min sites = 10. Sequences with homology to gene models in oomycetes genomes were identified by BLAST analysis against the NR database and aligned using MUSCLE [133]. For phylogenetic inference of the *CRN *genes, alignments were done using RevTrans [134] with the dialign-T algorithm. Molecular phylogenetic reconstructions were done using RAxML [135] version 7.0. Sequence logos were constructed on the basis of the RevTrans alignment using WebLogo [136].

### Comparative genomics analyses

In order to find substantial expansions and contractions of gene families observed in other eukaryotes, we used the PANTHER Classification System [49,137,138]. We first scored all predicted proteins from the *P. ultimum *genome against the PANTHER HMMs, and created a tab-delimited file with two columns: the *P. ultimum *protein identifier and the PANTHER HMM identifier from the top-scoring HMM (if E-value < 0.001). We created similar files for three *Phytophthora *genomes (*Ph. infestans*, *Ph. ramorum*, and *Ph. sojae*), and a diatom genome (*P. tricornutum*) for comparison. We removed protein families of probable viral origin or transposons (PTHR19446, PTHR10178, PTHR11439, PTHR23022, PTHR19303). This left 7,762 *P. ultimum *proteins in PANTHER families, 8,169 from *Ph. infestans*, 7,667 from *Ph. ramorum *and 7,701 from *Ph. sojae*. We then uploaded the tab-delimited files to the PANTHER Gene List Comparison Tool [137,139] and analyzed the list for under- and over-representation of genes with respect to molecular functions, biological processes, and pathways. For each class that was significantly different (Bonferroni-corrected *P *< 0.05) between *P. ultimum *and all of the *Phytophthora *genomes, we determined the protein family expansions or contractions that made the biggest contributions to these differences (Table 1). Finally, we determined likely gene duplication and loss events that generated the observed protein family expansions and contractions by building phylogenetic trees of each of these families using the 48 genomes included in the trees on the PANTHER website [140], in addition to the five stramenopile genomes above (*P. ultimum*, *Ph. infestans*, *Ph. ramorum*, *Ph. sojae*, *P. tricornutum*). Phylogenetic trees were constructed using the GIGA algorithm [141], which infers the timing of likely gene duplication events relative to speciation events, allowing the reconstruction of ancestral genome content and lineage-specific duplications and losses. Using v3 of the annotation (MAKER output without manual curation), *P. ultimum *genes orthologous to genes in *Ph. infestans*, *Ph. sojae *and *Ph. ramorum *were identified using PHRINGE ('Phylogenetic Resources for the Interpretation of Genomes') [103] in which the evolutionary relationships among all oomycete protein families are reconstructed.

### Carbohydrate utilization

Growth was compared on different media. Carbon sources were added to Minimal Media (MM; per liter: 0.5 g KH_2_PO_4_, 0.5 g K_2_HPO_4_, 4 × 10^-4 ^g MnSO^4^, 4 × 10^-4 ^g ZnSO_4_, 1.05 g NH_4_Cl, 6.8 ml 1M CaCl_2_·2H_2_O, 2 ml 1M MgSO_4_·7H_2_O, 4 × 10^-3 ^g FeSO_4 _and 1% (w/v) agarose) at the following concentrations: 1% (w/v) for cellulose, soluble starch, citrus pectin and birchwood xylan and 25 mM for D-glucose, D-fructose, D-xylose, cellobiose, sucrose and L-arabinose. The pH of the medium was adjusted to 6.0 and the medium was autoclaved at 121°C for 20 minutes. CaCl_2_, MgSO_4_, and monosaccharides were autoclaved separately from the rest of the medium and FeSO_4 _was sterile filtered (Whatman 0.2 μm millipore filter, Dassel, Germany). All of these components were added to the autoclaved medium before it solidified. The growth of *P. ultimum *DAOM BR144 was compared on the different media mentioned above; Minimal Media without a carbon source was used as the negative control in this experiment. The strain was initially grown on Potato Carrot Agar [142]. A small agar plug containing mycelium (1 mm diameter) was transferred from the edge of a vigorously growing 1-day-old colony to the center of the Petri dishes with the different media. The cultures were incubated in the dark at 21°C. Mycelium density and colony diameter were measured daily for the first 5 days and again after 7 days. Colony morphology pictures were taken, and pH was measured after 7 days. The growth test was conducted twice for each strain.

## Abbreviations

bp: base pairs; CBEL: cellulose-binding elicitor lectin; CRN" Crinkler; EC: extracellular; EST: expressed sequence tag; FIR: flanking non-coding region; GH: glycoside hydrolase; HMM: Hidden Markov Model; MAMP: microbial-associated molecular pattern; ORF: open reading frame; PAMP: pathogen-associated molecular pattern; RPKM: reads per kilobase of exon model per million mapped reads; YEB: yeast extract broth.

## Authors' contributions

CAL, NT, and CRB directed the project, performed analyses, and drafted the manuscript. GWB drafted the manuscript. HB, LC, EH, SR, GR, MT, JW, JLB, BD, SIF, CMMG, EG, FG, LG-B, NH, RHYJ, TK, HJGM, PM, VP, ST, SW, PvW, PMC, BH, FM, PDT, BMT, RPDV, and SK participated in the genome analysis and drafted the manuscript. JPH, CH, BRW, and MY conducted genomic/transcriptomic analyses and drafted the manuscript. MMZ isolated RNA, constructed cDNA libraries, participated in the genome analysis and drafted the manuscript. DB, JJ, HL, BM, DP, and JES conducted genomic/transcriptomic analyses. SF, JH, and JS performed genome sequencing. All authors read and approved the final manuscript.

## Supplementary Material

Additional file 1 **Supplemental methods and results**. Additional details on sequencing methods and analysis results citing methods or data from [147-166]. Click here for file

Additional file 2 **Supplemental Tables S1 to S11 providing detailed lists and analyses**. Click here for file

Additional file 3 **Supplemental Figures S1 to S16, supporting data analyses**. Click here for file

Additional file 4 **Multiple sequence alignment of oomycete proteins with similarity to *P. ultimum *Family 3 proteins**. Predicted secreted proteins (91) with similarity to Family 3 proteins from various oomycete species were aligned demonstrating the YxSL[KR] motif. Click here for file
