# Supplementary material for: Genome sequence of the necrotrophic plant pathogen Pythium ultimum reveals original pathogenicity mechanisms and effector repertoire
Source: Genome Biol. 2010 Jul 13;11(7):R73. doi: 10.1186/gb-2010-11-7-r73 (PMC2926784; doi:10.1186/gb-2010-11-7-r73)
Supplement: Additional file 1 — Supplemental methods and results. Additional details on sequencing methods and analysis results citing methods or data from [147-166]. [file gb-2010-11-7-r73-S1.doc]

**Additional Data File 1:** **Supplemental Methods and Results**

***P. ultimum* DAOM BR144 Genome Statistics**

The final *P. ultimum* DAOM BR144 genome assembly used for analysis consisted of 975 scaffolds that were comprised of 1,747 contigs with an average depth of coverage of ~8X. The N_50_ contig length is 124 kb and the N_50_ scaffold length is 773,464 bp. There are 772 scaffold gaps comprising 2.1Mb of N’s in the scaffolds which represent sequencing gaps. The GC percentage of the genome is 52.3% and based on the assembly, the genome size is estimated to be 42.8 Mb, comparable to genome sizes in two related *Phytophthora* species [27]. The variable base finding algorithm within Celera Assembler [108, 147, 148] was used to identify high quality reads in conflict with the consensus sequence. A variable base was called when there were a minimum of two reads with an average quality value greater than or equal to 21 or when there was a minimum of two reads and the sum of the top two quality values was greater than or equal to 60. We mapped a set of transcript assemblies derived from Sanger and 454-generated ESTs to the genome [31] to assess the representation of the genome in our final assembly. We were able to map 10,122 (94.6%) of the contigs and 19,930 (86.8%) of the singleton ESTs. If EST assemblies and singletons derived soley from Sanger sequencing were examined, 99.75% and 97.9%, respectively, could be aligned with the *P. ultimum* DAOM BR144 genome assembly.

A total of 122,926,977 reads representing 4.7 Gb of transcriptome sequence were generated from eight cDNA populations. Following quality filtering, 83,705,428 reads (3.2 Gb) remained. Using TopHat and Bowtie, 71,047,867 reads (85%) representing 2.7 Gb could be mapped to the DAOM BR144 genome, of which, 90% mapped uniquely to the genome. Transcript frequency ranged from 0 to 32,041 RPKM with the median ranging from 5-7.9 and a mean ranging from 79-85 across the eight cDNA samples. Using a cutoff value of 2.5 RPKM, 11,685 loci were expressed in at least one library, 7,622 loci were expressed in all eight libraries, and 1,021 loci were expressed only in a single library. Looking at the 100 highly expressed genes across the eight cDNA populations revealed high levels of expression for ribosomal proteins and other translational apparatus components, elicitins, ubiquitin, and genes of unknown function (see Table S8 in Additional Data File 2). Using a cutoff of > 2-fold (see Table S6 in Additional Data File 2), we were able to identify a number of genes that are differentially expressed among the different growth conditions.

**Additional *P. ultimum* DAOM BR144 Annotation Features**

Of transcripts with extremely long open reading frames, the longest is 21,357 bp (PYU1_G014053). Interestingly, homologs of large single exon *P. ultimum* genes tend to be intron rich in other eukaryotes. One example of this is midasin, which encodes a highly conserved ~600 kDa nuclear chaperone protein. The ortholog of this gene in *P. ultimum* is intronless, yet the orthologous gene contains 72 introns in *Arabidopsis thaliana* and 101 introns in *Mus musculus* (See Figure S11 in Additional Data File 3). In the three sequenced *Phytophthora* spp., homologs of midasin are present and contain few introns (maximum of 7). In *Saccharomyces cerevisiae*, however, this gene is encoded by a single exon, showing a striking similarity to *P. ultimum* considering the large evolutionary distance between these organisms (See Figure S11 in Additional Data File 3).

**Repetitive content in DAOM BR144**

A total of 7,722 repeats were identified by RepeatMasker as being simple (low complexity) repeats comprising ~1% of the genome by length whereas 1,714 were identified by the same approach as being of high complexity, also comprising ~1% of the genome (see Table S2 in Additional Data File 2). Additional repeats from the *P. ultimum* specific repeat library (1,693) were identified comprising ~2 % of the genome (see Table S2 in Additional Data File 2). We also identified another 1,686 repeats with homology to the MAKER internal transposable element protein-database, comprising ~3% of the genome. DNA methylases identified by protein domain analyses in the *P. ultimum* genome are PYU1_T000288, T000595, T001212, T001274, T002163, T002828, T003813, T003889, T006956, T007064, T007474, T007739, T008329, T008491, T009231, T009467, T009760, T009763, T012099, T014901, T015322.

***P. ultimum* mitochondrion**

Several putative ORFs are shared with other oomycetes (ymf98, ymf99, ymf100, ymf101, and orf32) whereas two others (orf169, orf170) are unique to *P. ultimum*. Prior analysis of the shared putative ORFs suggested that these were functional genes due to the low rate of non-synonymous compared to synonymous substitutions in *Phytophthora* [48]. With respect to the tRNAs, there are 42 total tRNAs that encode 19 different amino acids with 17 present in each arm of the IR (34 total) and 8 in the large unique region.

**Additional Enzymes involved in Carbohydrate Metabolism**

The *P. ultimum* genome encodes large numbers of enzymes involved in carbohydrate degradation and modification. Results from the annotation point at a strong capacity to degrade β‐1,3- and β‐1,4-glucans. These enzymes may be used either on *P. ultimum*’s own cell wall metabolism or directed for plant carbohydrate degradation. Furthermore, a large set of β-1,3-glucan synthases within the glycosyl transferase (GT) family GT48 were identified. GT48s are likely involved in synthesis of cell wall β-glucans and in the metabolism of mycolaminaran. Five putative GT48 β‐1,3-glucan synthases were 2-5 fold up-regulated when *P. ultimum* was grown in the presence of germinating *Arabidopsis* seeds. However, the majority of the other genes related to β‐1,3-glucans were not differentially expressed under the conditions assayed, indicating that they may be part of the oomycetes own β‐1,3-glucan metabolism.

Ten putative β-glucosidases are found in family GH30, a family with typically fewer members among Fungi, the large numbers in this family suggest an important role for these enzymes in degradation of carbohydrates. An ortholog of this gene was studied for *Ph. infestans* (BGX1), but its function remains poorly understood [149]. More β-glucan degrading enzymes were found within families GH1, GH2, and GH3. Genes belonging to these families may be involved in either the metabolism of cellulose, β-1,3-glucan, mycolaminaran or other unidentified components. Five genes (PYU1_G003466; PYU1_G013445; PYU1_G013457; PYU1_G013458; PYU1_G013459) within family GH1 were up-regulated (2-8 fold) when *P. ultimum* was grown in the presence of Arabidopsis seeds; for all five proteins a C-terminal transmembrane region and signal peptide were predicted and thus, they may be associated with oomycete cell wall metabolism. Four genes belonging to the GH2 (PYU1_G013467) and GH3 (PYU1_G008948, PYU1_G012504, PYU1_G012853) families presenting signal peptides were up-regulated (2-15 fold) in the presence of Arabidopsis seeds and may play a role in plant carbohydrate degradation.

The low chitin content of *P. ultimum* cell walls [150] is consistent with a very limited number of enzymes dedicated to chitin metabolism, its genome contains a single family GT2 chitin synthase and three GH18 enzymes distantly related to chitinases, while no GH20 candidate b-N-acetylglucosaminidase or any family CBM18 chitin-binding modules are identified. The chitin synthase gene was up-regulated (15 fold) when *P. ultimum* was incubated with *Arabidopsis* seeds. The reason for this is unknown, although the higher expression may compensate for damage caused to the cell wall by plant chitinases, or may indicate that the *P. ultimum* cell wall is modified during the infection process.

We detected eight expansin related-genes and these may cause loosening and extension of cell walls by disrupting non-covalent bonding between cellulose microfibrils and matrix glucans [151] (Figure 4). Absence of glycogen-related enzymes in the genome (GHs and GTs) strongly suggests that glycogen is not used as an energy and carbon reserve in *P. ultimum.* The genome encodes ten genes belonging to families GH13, GH15 and GH31, most likely used for saccharification of starch from the host plant. Three proteins (two GH13 and one GH31) contain a peptide signal and N-terminal starch-binding CBM25 module and two GH15 enzymes contain a peptide signal and N-terminal CBM21 starch-binding module. *P. ultimum* encodes a family GH32 enzyme related to invertases.

**Utilization of carbon sources**

Carbon source growth assays are an excellent way to provide experimental support for the functional annotation of carbohydrate-active enzymes [152, 153]. The results from the carbon source utilization experiment are summarized in Table 3 and see Figure S10 in Additional Data File 3. The negative control showed very sparse growth, hyphae of only several days old were devoid of cytoplasm, and quickly formed septae. The residual growth is likely caused by nutrients carried over in the inoculation plug, or the presence of trace amounts of carbon in the other components of the medium. Differences in mycelium density and colony morphology between substrates only emerged after a lag period of approximately 2 days. Of the monomeric sugars, D-glucose and D-fructose resulted in good growth (Table 3). Growth on the other monomeric sugars tested was similar to the negative control. On media that supported good growth of *P. ultimum*, a sharp decrease of the pH of the medium was observed. This effect has been previously described, and was found to be linked to the use of ammonium as a nitrogen source [154]. The data from the carbon source utilization experiment shows only limited growth on medium with citrus pectin as the sole carbon source. This supports the hypothesis that pectin degrading enzymes in *P. ultimum* are produced as a means to facilitate penetration into the intracellular pectin-rich tissue, while use of pectin as a carbon source plays no significant role. Similarly, despite the presence of putative cellulases, no growth was obtained on cellulose as a unique carbon source. The disaccharide cellobiose however was used very efficiently. This suggests that in *P. ultimum,* plant cell wall degrading cellulases are expressed only to permit access to the host cell in a pathogenic context, rather than for plant cell-wall saccharification. This is in agreement with a study by Chérif et al. [74] who demonstrated, using cytochemical labeling, that cellulose degradation was only localized to the path of infection. Other results of the carbon source utilization experiment are consistent with the results from the annotation: no growth was obtained when xylan was used as a carbon source, confirming the absence of xylan degrading enzymes, while the presence of a starch and sucrose degrading capacity is confirmed by good growth of *P. ultimum* on these substrates as sole carbon sources.

**Detection of *P. ultimum* by the host**

The transglutaminase GP42 cell wall glycoprotein of *Ph. sojae* contains a 13 amino acid stretch (Pep-13) able to stimulate defense responses on parsley and potato. Transglutaminase with highly conserved Pep-13 motifs have been detected in all *Phytophthora* species [66]. Amino acid residues W2 and P5 are important for phytoalexin induction and the nature and spacing between N3 and Q4 are important for Pep-13 receptor binding [66]. Four putative TGAses genes were detected in the *P. ultimum* genome encoding predicted proteins which contain the Pep13 sequence harboring the essential amino acid residues W2, Q4, P5, N3 except in PYU1_T011421 which shows an amino acid substitution for the Q4 residue (see Figure S12 in Additional Data File 3). This suggests that *P. ultimum* TGAses would be able to induce plant defense.

We also examined the presence of Cellulose-Binding Elicitor Lectin (CBEL) genes. A CBEL protein, initially discovered in *Phytophthora parasitica*, is an associated cell surface glycoprotein without catalytic activity playing a role in adhesion to host wall components [155]. The Carbohydrate-Binding Module 1 (CBM_1; IPR000254) domain found in CBEL proteins is essential for the binding to cellulose and sufficient to trigger innate immunity on various plants such as *Arabidopsis* and tobacco [67]. Another domain found in CBEL proteins is the so-called N/Apple PAN domain (IPR000177) also detected in virulence proteins of Apicomplexa. CBELs or CBEL-like genes (proteins containing CBM_1 but not N/ApplePAN domain) are widely distributed among oomycetes, being present in species phylogenically distinct from *Phytophthora* such as *A. euteiches* [64, 156]. Three *P. ultimum* genes (PYU1_T006785, PYU1_T009917, PYU1_T002920) encoding the canonical version of CBEL were detected. However, 13 genes coding modified versions of CBEL sequences, corresponding to either one CBM_1 associated to one N/Apple domain (10 genes models) or a repetition of CBM_1s (3 gene models; PYU1_T003169, PYU1_T003783; PYU1_T003171) were also identified, as well as genes coding glycosylhydrolases with CBM_1 domains. Interestingly, 20 predicted protein sequences were detected having similarity with CBEL centered only on the N/Apple PAN domain (IPR000177 and IPR003014). Multiple alignment of the *P. ultimum* sequences with the CBEL CBM_1 domains revealed a conserved pattern centered on a conserved core of four cysteine residues, and aromatic residues known to be important for binding the carbohydrate substrate (see Figure S13 in Additional Data File 3).

**Protease inhibitors**

Pathogen protease inhibitors are known to interact and inhibit plant proteases implicated in counter-defense [157, 158]. The tomato and potato pathogen *Ph. infestans* secretes two major structural classes of protease inhibitor proteins: (1) Kazal-like serine protease inhibitors (EPIs) and (2) Cystatin-like cysteine protease inhibitors (EPICs) [159, 160]. Both structural classes have been described in two other plant and one animal pathogen oomycete species: *Plasmopara halstedii*, causal agent of downy mildew of sunflower [161], *A. euteiches*, legume pathogen [64], and the fish pathogen *Saprolegnia parasitica* [156].

To investigate protease inhibitor encoding genes in *P. ultimum,* we performed a BLASTP search using *Ph. infestans* protease inhibitor proteins as the query. We also did a TBLASTN search to confirm no additional gene models from the scaffolds with similarity to protease inhibitors were present. We identified 37 proteins with similarity to *Ph. infestans* Kazal-like serine protease inhibitors that clustered in Families 021, 041 and 048 (see Table S9 in Additional Data File 2): 28 secreted and 9 non-secreted proteins (4 without signal peptide and 5 with signal peptide and transmembrane domains). Sequence alignment to those known oomycete Kazal-like protease inhibitors showed conservation of the six cysteines backbone and the active site P1 (see Figure S14 in Additional Data File 3). We also identified six proteins with similarity to *Ph. infestans* cystatin-like cysteine protease inhibitors in Family 100 with the exception of one protein (see Table S10 in Additional Data File 2): 3 secreted and 3 non-secreted proteins (with signal peptide and transmembrane domains). Sequence alignment of their putative cystatin-like inhibitor domains highlights the conserved amino acids in the N-terminal trunk and loop1 domain (see Figure S15 in Additional Data File 3). These findings together suggest that protease inhibitors are common features of oomycetes.

**Phospholipase Gene Family Analyses**

The phospholipase D (PLD) reduction is only found for two out of the six PLD subfamilies (PLD-likes and sPLD-likes) that were previously recognized in oomycetes [162]. Each PLD subfamily is still represented at least once in the genome. As found for other oomycetes, *P. ultimum* lacks a gene encoding a phospholipase C [27].

**Responses to the Environment**

As a soil-borne organism, *P. ultimum* is influenced by fluctuation in oxygen concentrations, particularly in water-saturated soils where it can cause significant root rot damage. A total of 78 genes were up-regulated at least five fold during hypoxia (see Table S6 in Additional Data File 2). PYU1_G013247 encodes a cytochrome P450 that was expressed 36 times more than the control and is a member of a superfamily that is related to the oxidation of a wide array of different substrates [163]. The genes encoding NADH:flavin oxidoreductase/NADH oxidase and alcohol dehydrogenase, which are also involved with anaerobic fermentation, were up-regulated eight fold or more.

*P. ultimum* has a wide range of growth temperature and its 25-30 ^o^C optimum growth range is surprisingly high. A total of 152 genes were up-regulated at least five fold when *P. ultimum* was subjected to heat stress (35 ^o^C) including genes encoding for molecular chaperones, alcohol dehydrogenases, thioredoxins, elicitin-like proteins, and a calcium binding protein. Genes coding for trehalose-phosphatase were also up-regulated (up to 10 fold) during heat stress, but not under the other abiotic conditions tested. In yeast and other organisms, trehalose plays a role in osmotic, heat and desiccation stress tolerance [164, 165]. Genes up-regulated (5-62 fold) when *P. ultimum* was subjected to cold stress (0 ^o^C) included the serine peptidases S59 and S8/S53, chloroperoxidase, catalase-peroxidase, and many unknown proteins (usually smaller than 200 amino acids). Many copies of ubiquitin-related genes were also up-regulated (5-18 fold). However, these were distinct from those up-regulated under mefenoxam treatment.

**Comparison with diatoms**

Plastids were likely lost multiple times in the heterokont lineage [1, 4] and Tyler *et al* [27] showed numerous genes of likely red algal origin in the *Phytophthora* genomes, in common with the heterokont algae, *Thalassiosira pseudonana* (a diatom). Analysis of the genome of *T. pseudonana* showed that a high proportion of genes had homology with proteins of *A. thaliana* [5]. Bowler *et al*. [6] also showed that many genes of the diatom *Phaeodactylum tricornutum* had homology to plants as well as green and red algae. Mustapha *et al*. [166] established that approximately 2,500 diatom genes had a green or red algae origin in either *T. pseudonana* or *Phaeodactylum tricornutum*. These two sets of endosymbiotic genes were compared with the sequenced oomycete genomes (see Table S11 in Additional Data File 2). Out of the 1,757 and 1,862 green algae derived genes for *Phaeodactylum* and *Thalassiosira*, respectively, over 500 (501/584) were found in all genomes of *P. ultimum* and *Phytophthora*. Figure S16 in Additional Data File 3 shows the results for *P. ultimum*, *Ph. infestans* and *Ph. ramorum* but results are very similar with the other two possible Venn diagrams if we use *Ph. sojae*. The number of genes of red algae origin in diatoms (Figure S16 in Additional Data File 3) is much lower possibly because the genome of *Cyanidioschyzon merolae*, the red alga used for comparison, has about 20 % the number of proteins of the green algae genomes that were used [166]. About 90 % of the total number of homologous genes found from this set were shared between *P. ultimum* and *Phytophthora*, indicating that these were probably core genes with essential functions (Figure S16B). The number of genes of green algae origin that were unique to *P. ultimum* or unique to *Phytophthora* were about the same (37±1). There were 11 genes of red algae origin found in all *Phytophthora* but only five found only in *P. ultimum*. The red algae Phosphoribosylamidoimidazole-succinocarboxamide (SAICAR) synthase reported in *Phytophthora* [27] was found in all *Phytophthora* spp. but was lost in *P. ultimum* whereas the thiamine-phosphate pyrophosphorylase was only found in *P. ultimum*. The two genes with high similarity to cyanobacteria used for phylogeny [27] were also present in *P. ultimum*.
