# Supplementary material for: Genome sequence of the necrotrophic plant pathogen Pythium ultimum reveals original pathogenicity mechanisms and effector repertoire
Source: Genome Biol. 2010 Jul 13;11(7):R73. doi: 10.1186/gb-2010-11-7-r73 (PMC2926784; doi:10.1186/gb-2010-11-7-r73)
Supplement: Additional file 3 — Supplemental Figures S1 to S16, supporting data analyses. [file gb-2010-11-7-r73-S3.pdf]

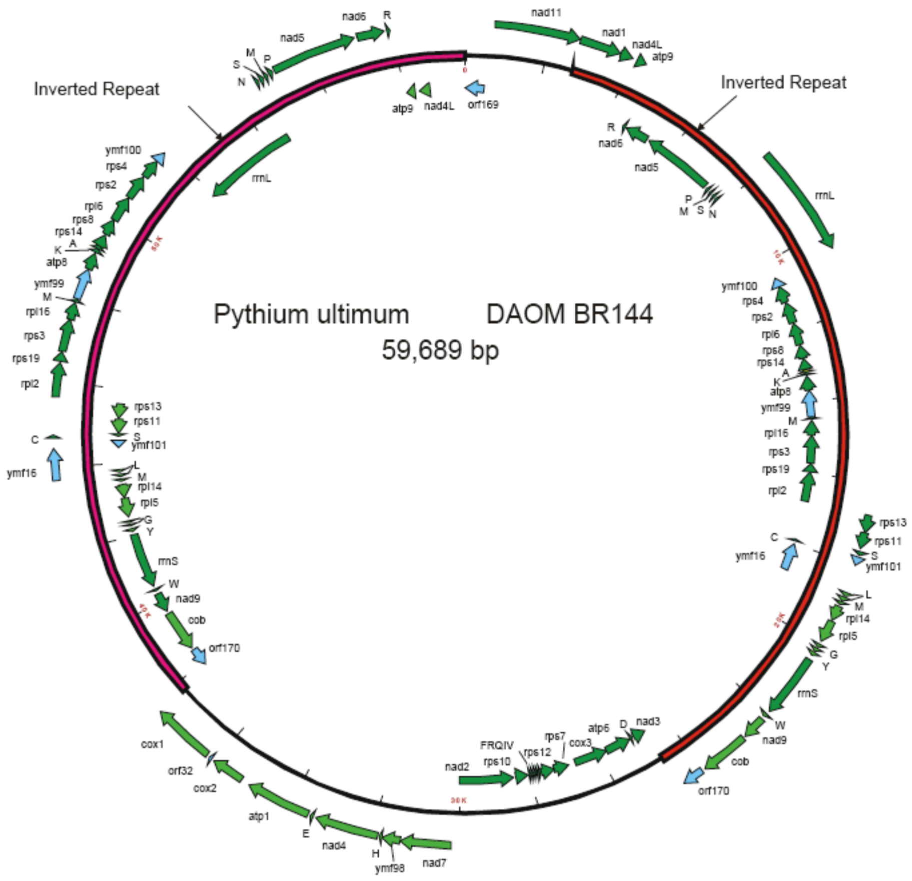

Figure S1: Mitochondrial gene map for *Pythium ultimum* BR144. Arrows indicate transcriptional orientation, clockwise for the outer row and counterclockwise for the inner row, with green representing coding regions and blue representing other unidentified ORFs. Genes for transfer RNAs are designated by the one-letter code for the corresponding amino acid. The position of the inverted repeat is indicated by the thicker vertical red line. The designation of "ymf" indicates unassigned ORFs that are conserved in the genus whereas "ORF" indicates unassigned ORFs of this number of predicted amino acids without obvious homologs.

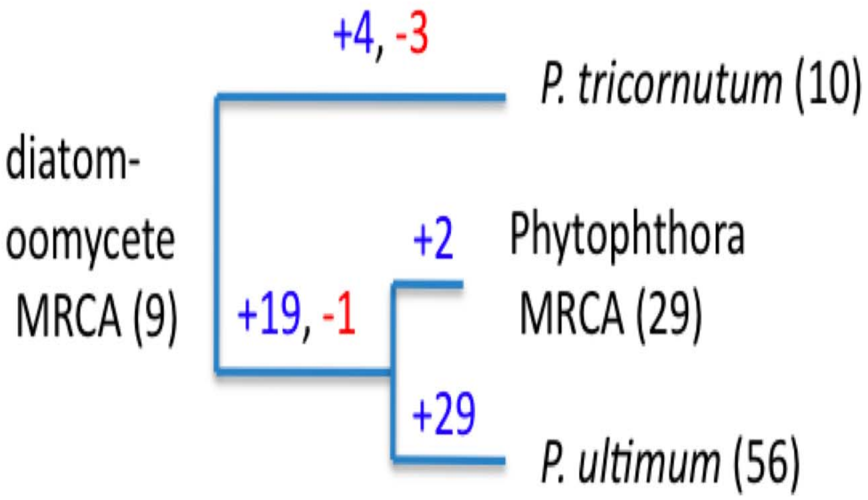

Figure S2. Phylogenetic tree cartoon showing the expansion of the HECT E3 ubiquitin ligase family from nine genes in the diatom-oomycete common ancestor to 56 in *P. ultimum*. There have been two periods of rapid expansion of this family, one between the diatom *Phaeodactylum tricornutum*, the stramenopile common ancestor (18 genes gained, one lost), and another in the *Pythium* lineage since the divergence from *Phytophthora* (29 genes gained total). Branches are not to scale. MRCA: Most Recent Common Ancestor.

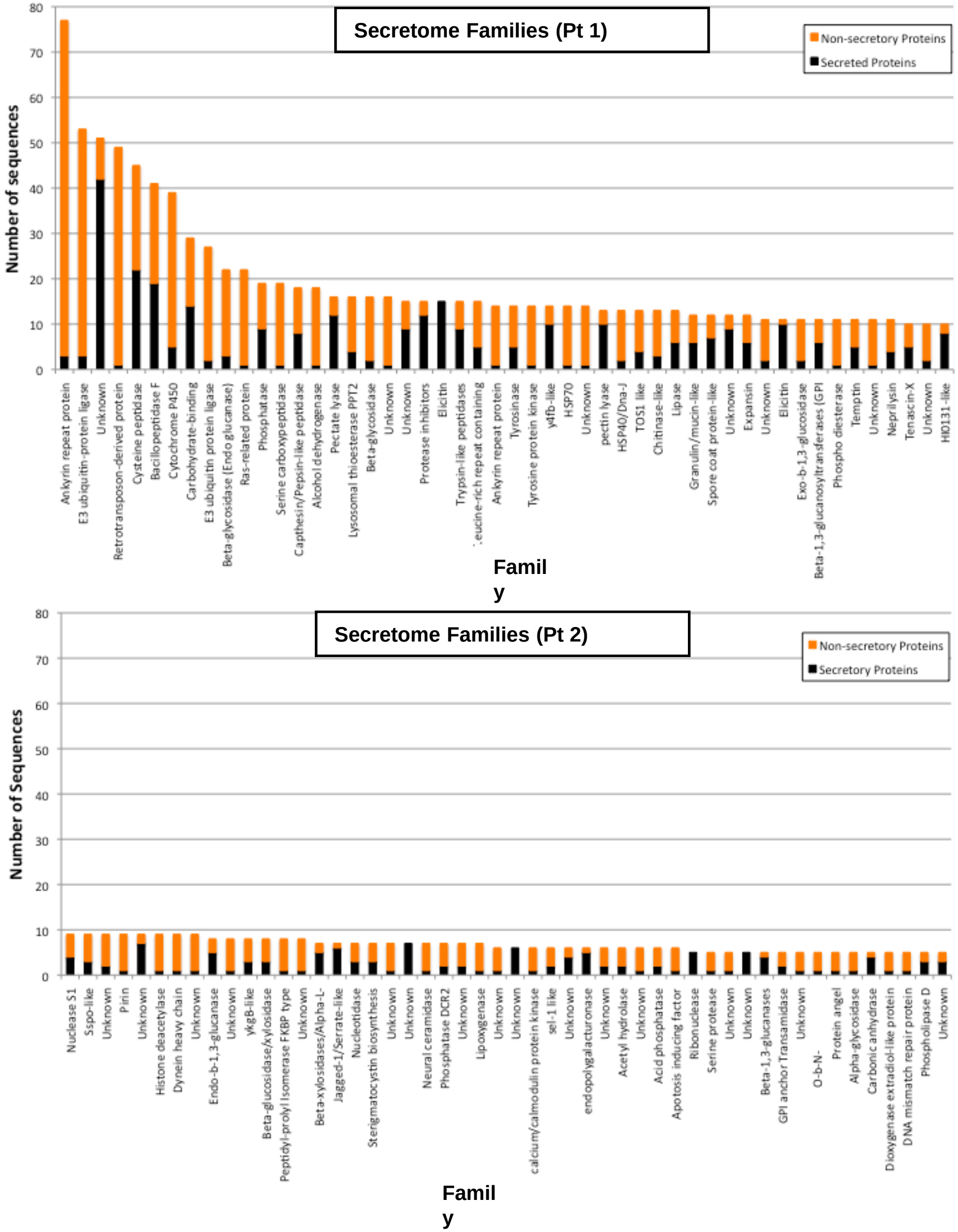

Figure S3. Distribution of secretory proteins and non-secretory proteins in the family clusters that contains at least one secretory protein in *P. ultimum* proteome.

A

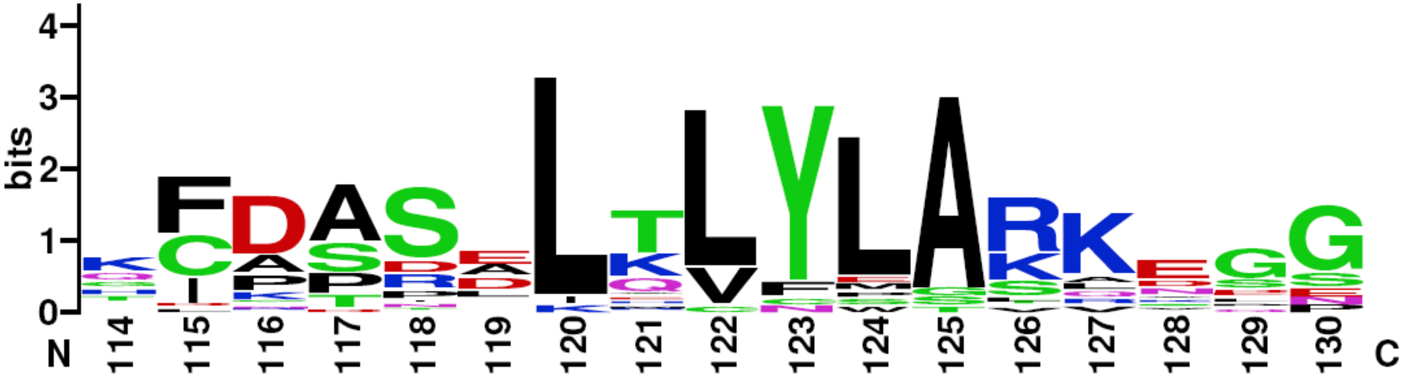

B

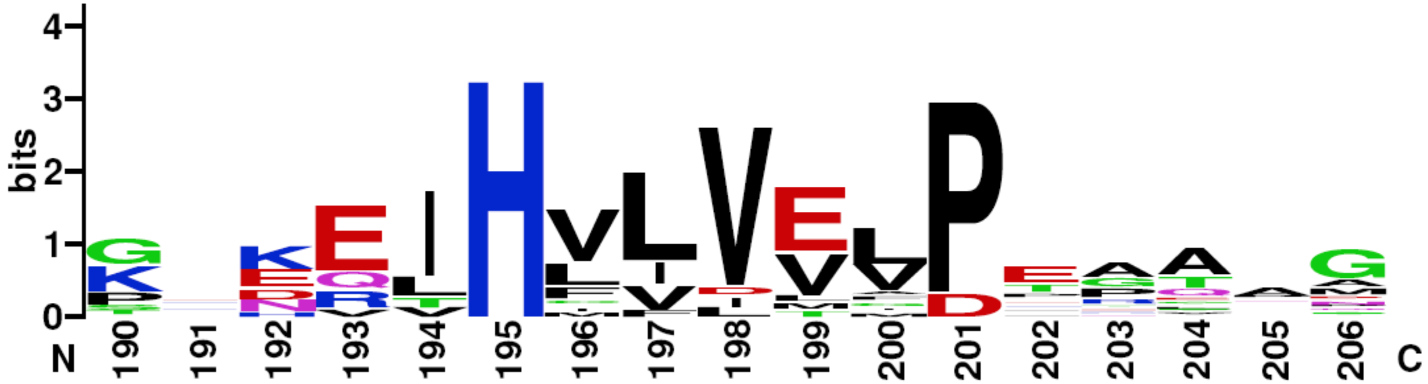

Figure S4. Sequence conservation in CRN-like predicted proteins of *P. ultimum*. Sequence logos were generated from alignments of the N-terminal domain of 26 CRN-like predicted proteins in *P. ultimum*. These reveal a conserved LxLYLAR/K motif (A) which is similar to the LxLFLAK motif found in the LFLAK domain of validated CRNs in *Ph. infestans*. The second motif (B), which is characteristic for the majority of CRNs and is found as a recombination site at the end of the DWL domain, can also be found in several CRN-like predicted proteins in *P. ultimum* with a consensus of HVLVxxP in those CRNs bearing an exact representation of the LxLLAR/K motif. Please note that the numbers in the sequence logo are referring to the corresponding positions in the alignment and thus differ largely from the average position of the motifs.

Figure S5. Phylogenetic reconstruction (Minimum Evolution) of the *crn*-like genes predicted in *P. ultimum*. The validated CRNs from *Ph. infestans* are given in red, predicted CRN-like proteins with some conservation of the recombination site (to a minimum of HxxVxxP) are given in green. Those CRN-like proteins of *P. ultimum* predicted to be secreted with significant support in SignalP v.2 are boxed.

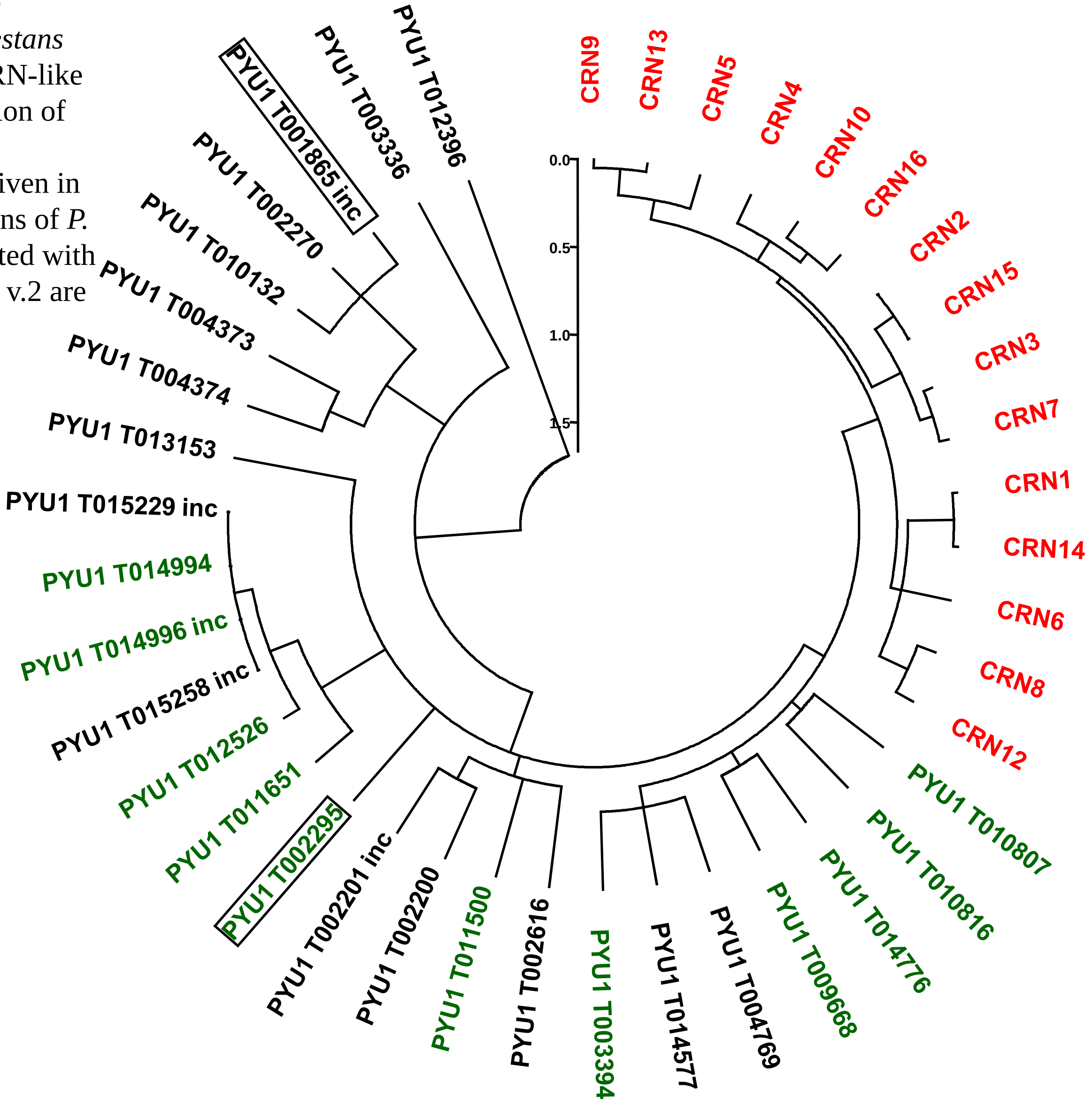

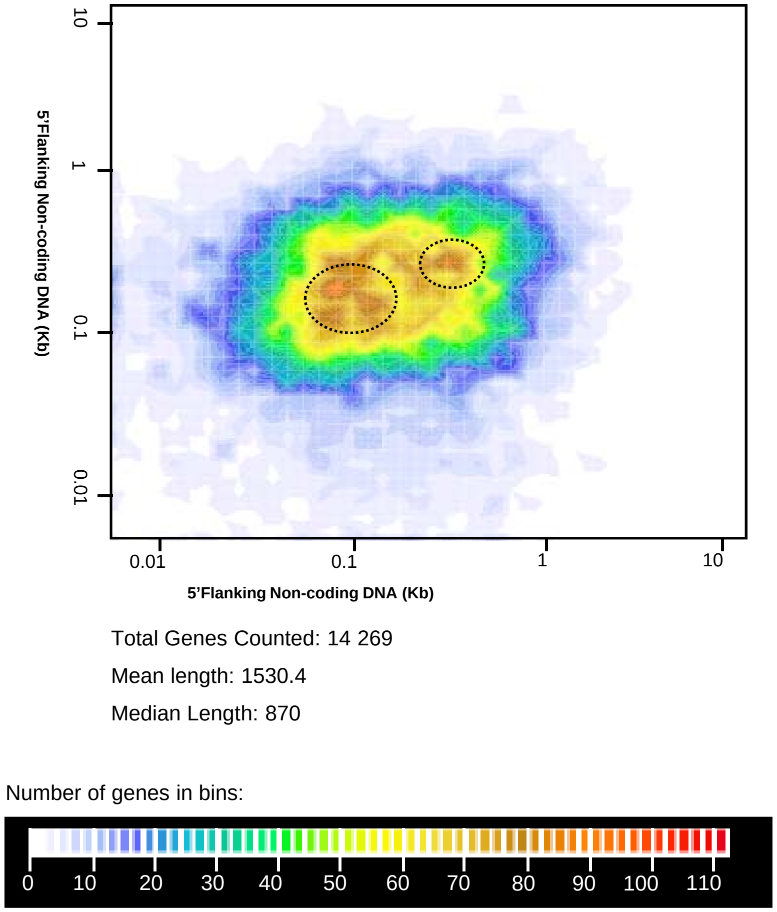

Figure S6. Organization of *P. ultimum* genome analyzed using flanking non-coding DNA regions (FIRs) length. All genes of the *P. ultimum* genome for which flanking non-coding DNA regions are determined (14,269) are sorted into 2D bins depending on the length of the 5' flanking region (X axis) and 3' flanking region (Y axis). This representation described in Haas et al. [28] allows visualizing the effect of transposon activity on global genome organization. As opposed to *Ph. infestans*, the distribution of *P. ultimum* FIRs is globally unimodal. However, the distribution of the main set of genes shows two distinct local maxima (dotted circles), that is distinctive from most of the genomes analyzed to date.

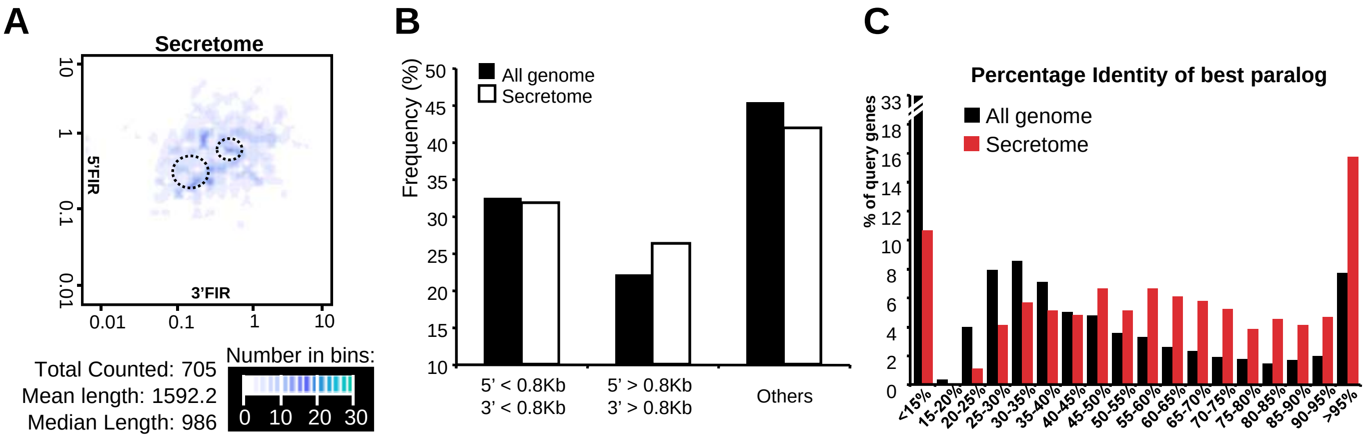

Figure S7. ***P. ultimum*** **secretome genes show longer flanking non-coding DNA regions and higher rate of recent duplication events. (A)** Secretome genes of the *P. ultimum* genome for which flanking non-coding DNA regions are determined (705) are sorted like in Figure S6. **(B)** Secretome genes more frequently have long (> 0.8Kb) flanking non-coding DNA region than average. **(C)** Secretome genes show a higher rate of highly identical (>95%) paralogs than the whole genome suggesting higher frequency of recent duplication events within the secretome.

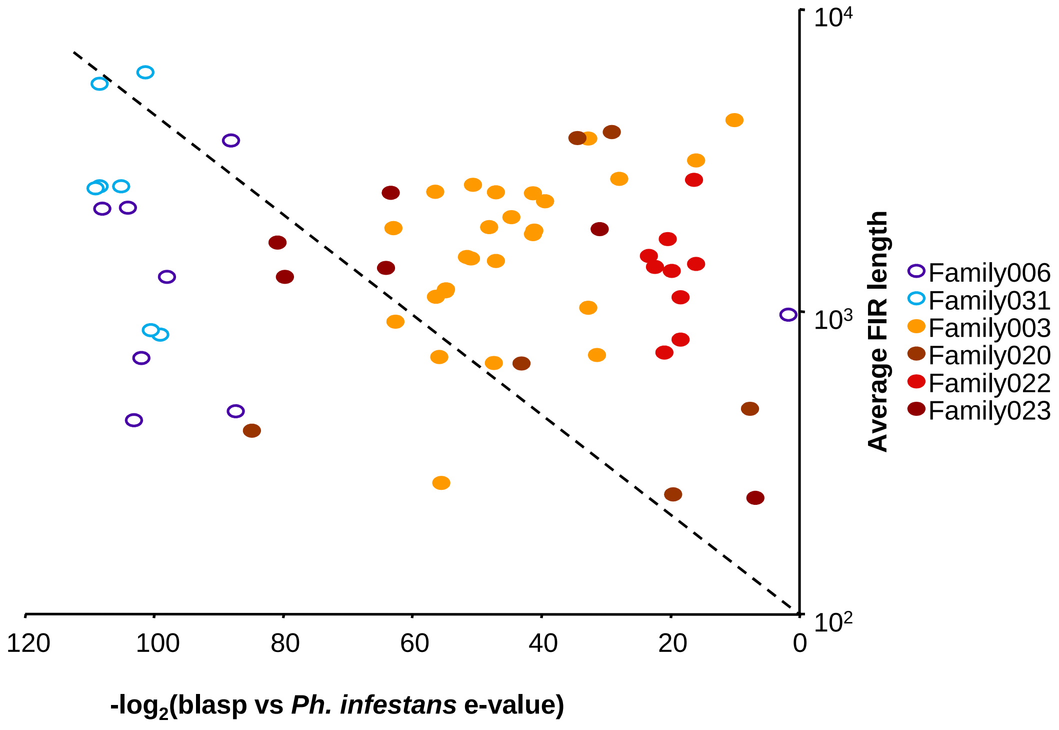

Figure S8. Example of the sorting process applied to secretome families based on gene conservation, length of flanking non-coding DNA regions and duplication rate. Genes from 6 families are plotted according to the -log2 (BlastP E-value against *Ph. infestans* proteome) reflecting protein conservation; and the average length of flanking non-coding DNA regions. A higher number of genes in family is indicative of higher duplication rate. Empty dots represent genes from families with frequent high conservation and short flanking non-coding DNA regions.

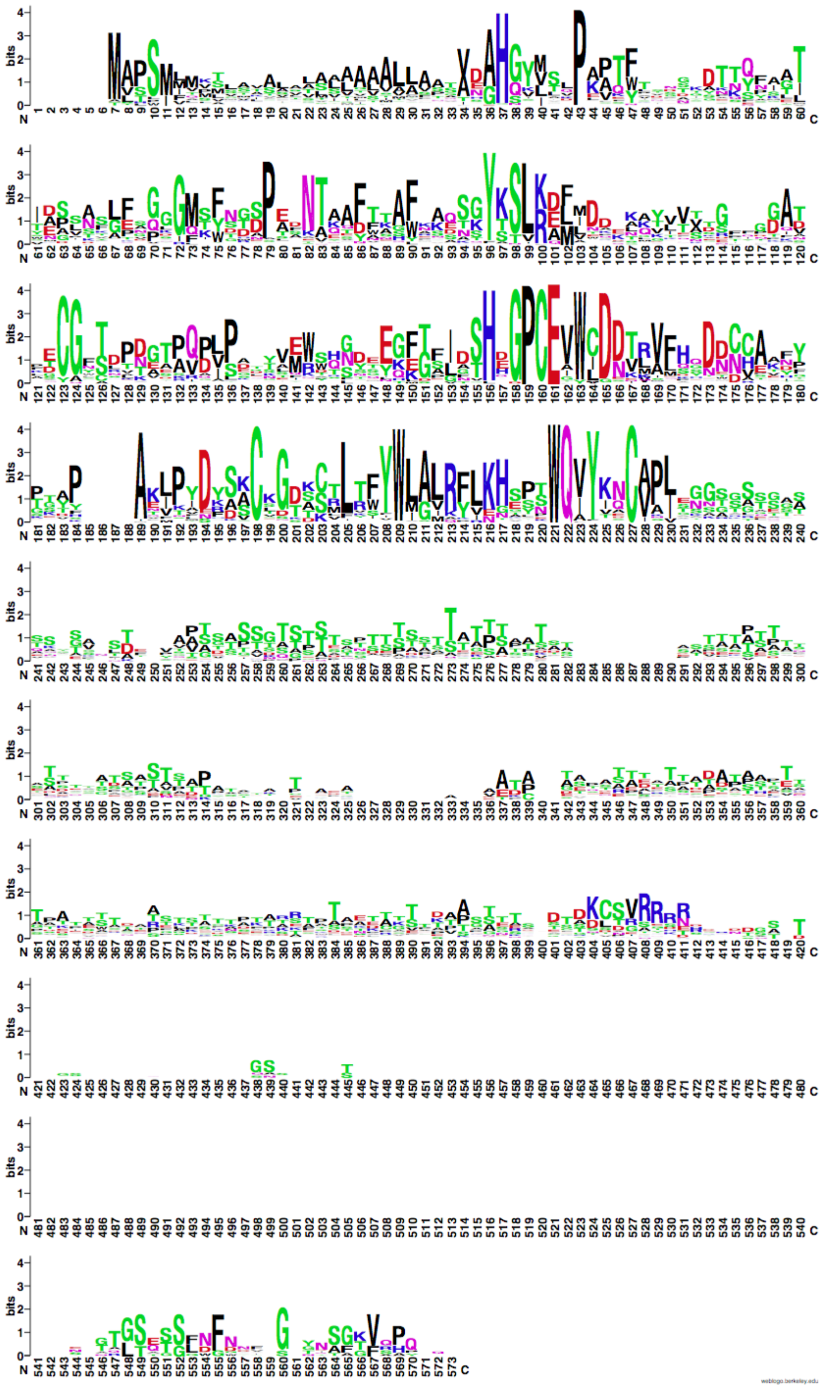

Figure S9. Full length weblogo generated using 91 secreted YxSL[KR] proteins from *P. ultimum*, *Ph. sojae*, *Ph. ramorum*, *Ph. infestans* and *A. euteiches*.

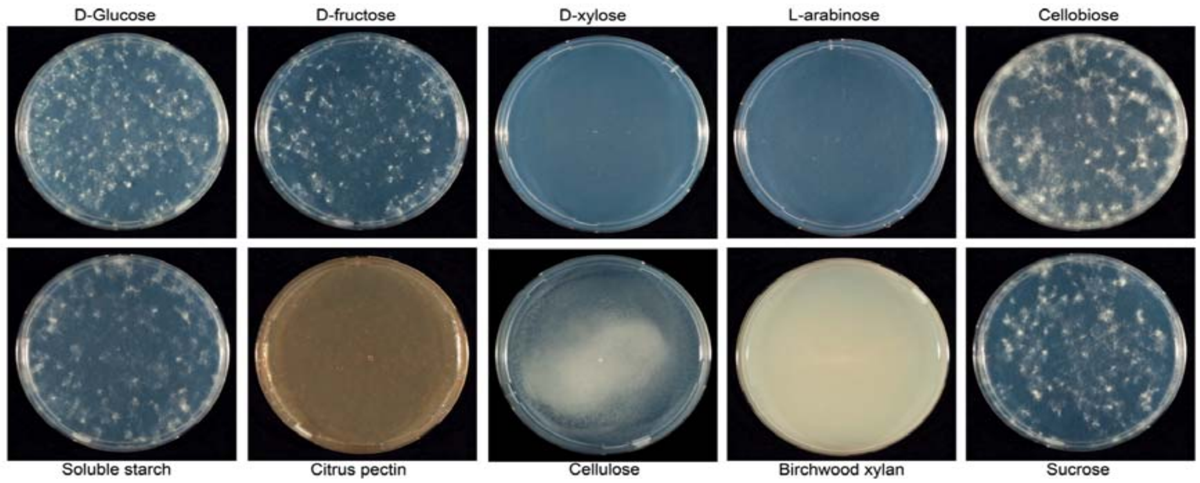

Figure S10. Carbohydrate utilization by *P. ultimum* strain DAOM BR144. *In vitro* growth of *P. ultimum* strain DAOM BR144 on different carbon sources. Concentrations for cellulose, soluble starch, citrus pectin and birchwood xylan were 1% (w/v) and 25 mM for D-glucose, D-fructose, D-xylose, cellobiose, sucrose and L-arabinose.

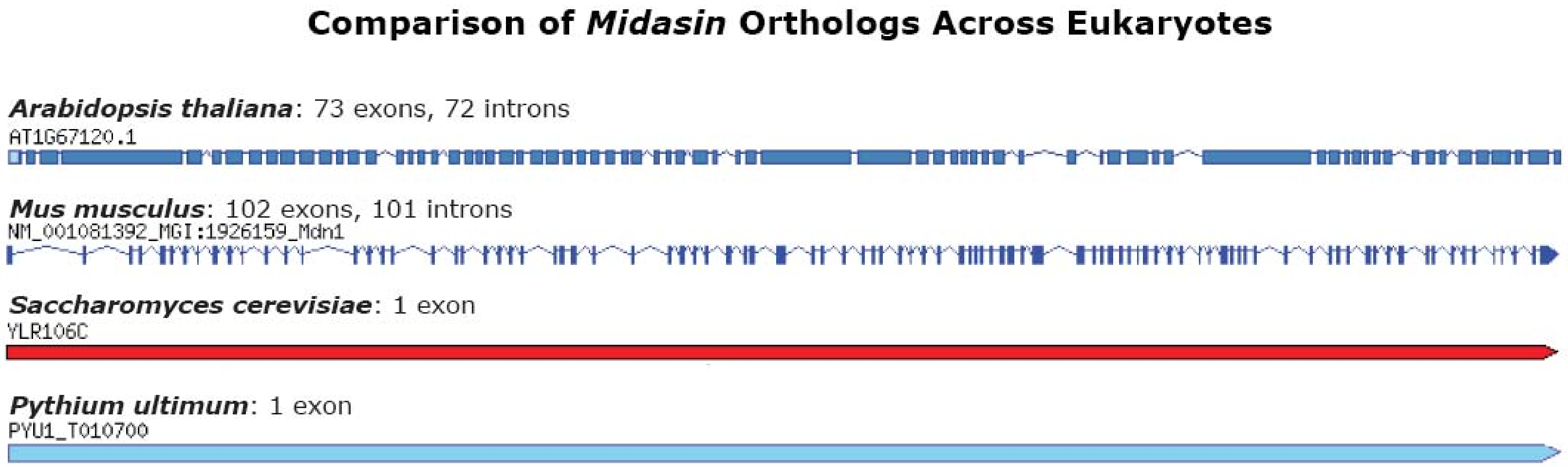

Figure S11. Long single exon genes in the *P. ultimum* genome. Many genes in *P. ultimum* are large single exon genes. However, orthologs of these single exon genes in other eukaryotic organisms tend to be intron rich, suggesting a possible evolutionary trend in *P. ultimum* for intron loss. The example in the figure shows orthologs of the gene *midasin*, which encodes a highly conserved ~600 kDa nuclear chaperone protein. Orthologs in both *M. musculus* and *A. thaliana* are intron rich; however, orthologs in *S. cerevisiae* and *P. ultimum* are both encoded by a large single exon. The similarity in gene stucture between *S. crevisiae* and *P. ultimum* is surprising given the evolutionary distance between organisms (Oomycetes are equidistant from plants, animals, and fungi) and suggests that intron loss may be a convergent adaptation related to a similar lifestyle.

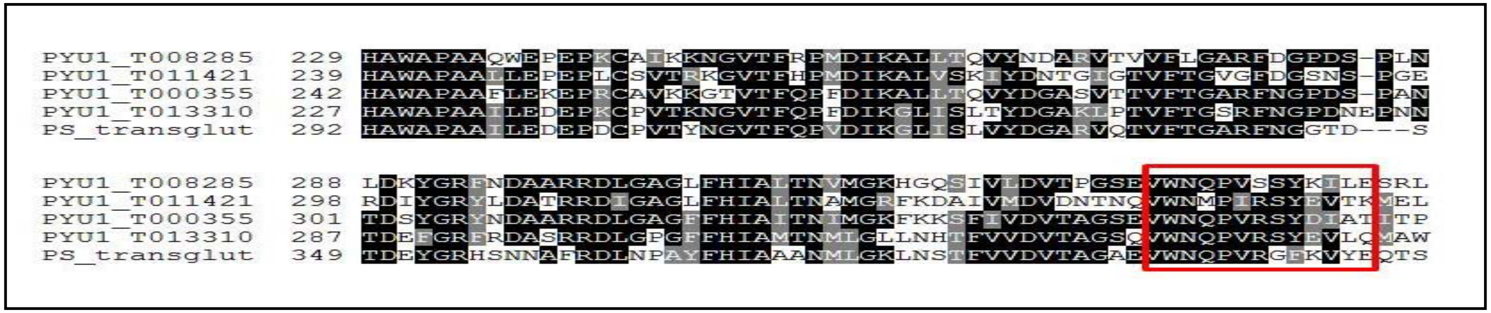

Figure S12. Multiple alignment of partial *P. ultimum* transglutaminase predicted amino acid sequences with the *Ph. sojae* protein. Red box indicates the Pep13 motif.

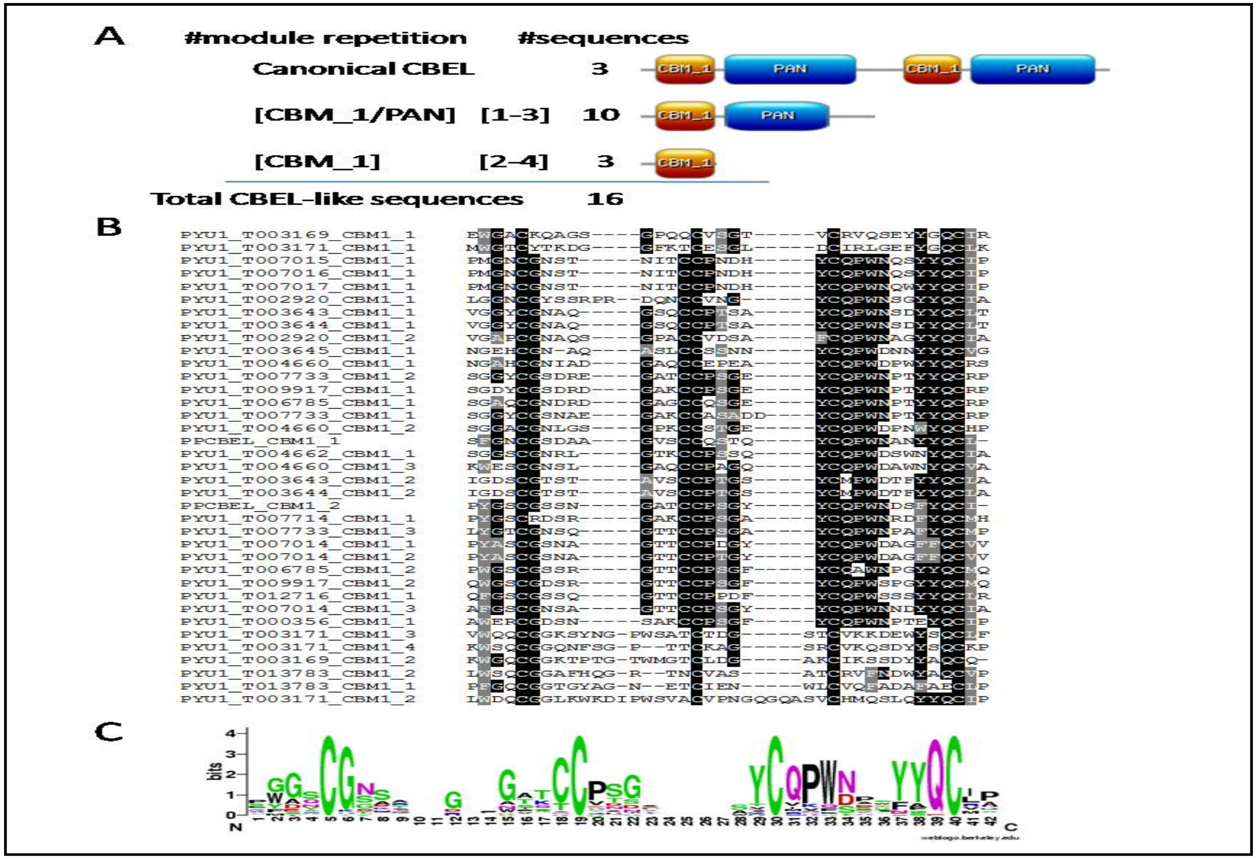

Figure S13. CBEL-like proteins in *P. ultimum* genome. A) The canonical version of the cell surface CBEL protein from *Phytophthora* is represented. The protein CBEL contains two closely associated domains, a carbohydrate-binding domain cellulose (CBM_1) and an N/apple PAN domain (PAN). CBEL-like sequences harboring a CBM_1 domain in association with various domains are illustrated. The number of domains repetitions is indicated in brackets and the number of genes with the corresponding domain organization is notified on the left part of the figure. Proteins containing CBM_1 with glycosylhydrolases activity are not illustrated in this section of the manuscript. B). Multiple alignment of CBM_1 domains present in CBEL-like proteins of *P. ultimum* with the domains of *P. parasitica* CBEL. Multiple domains originating from the same sequence are marked with the numbers 1, 2 or 3 at the end of the sequence name . The glutamine and aromatic residues that are known to be important for binding the carbohydrate substrate are shown by asterisks. C). Consensus sequence pattern of *P. ultimum* CBM_1 calculated using WebLogo showing the most conserved amino acids residues.

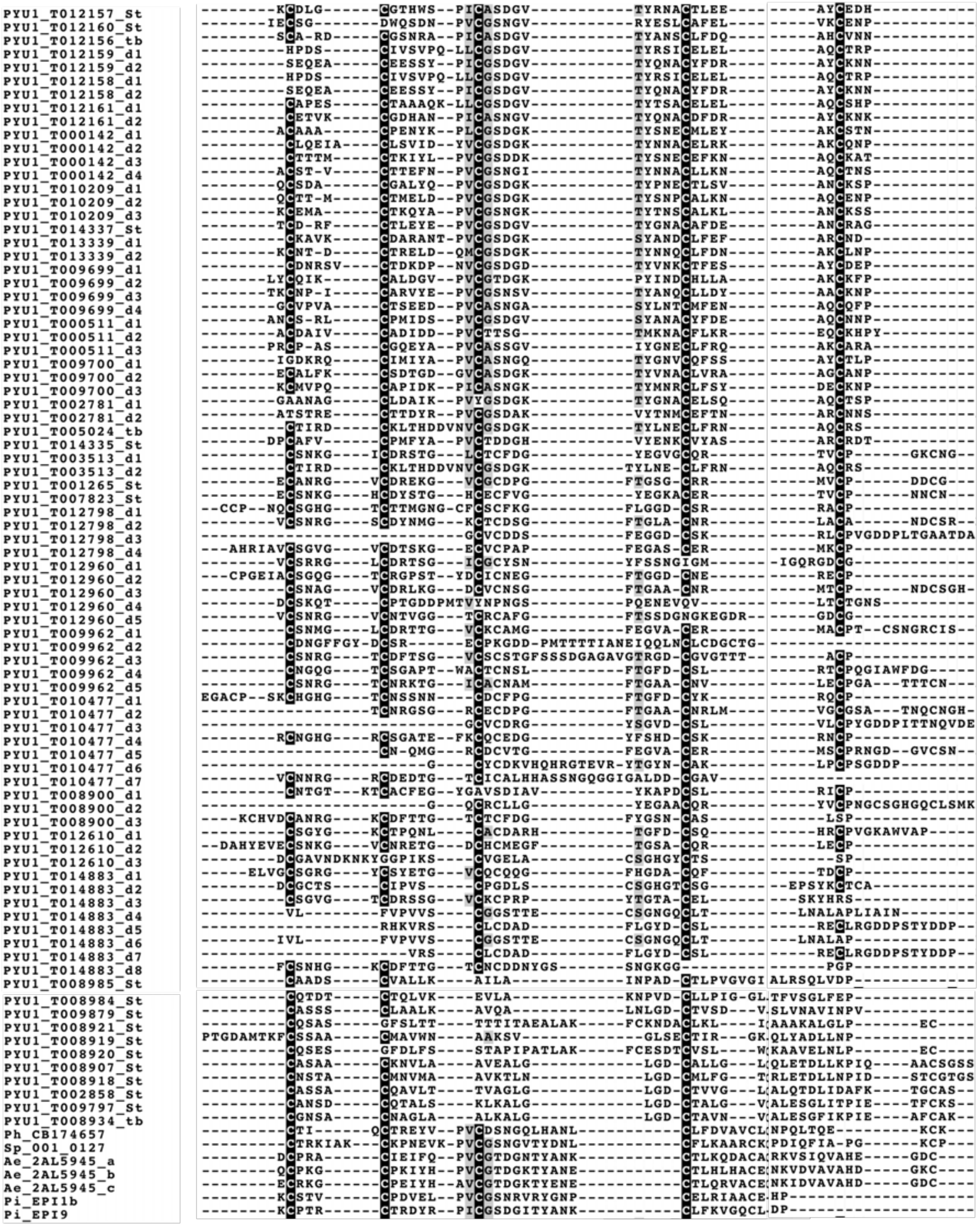

Figure S14. A family of serine-like protease inhibitors predicted in *P. ultimum* genome. Sequence alignment of 86 putative Kazal-like inhibitor domains from thirty seven predicted proteins in *P. ultimum* with other oomycete Kazal-like protease inhibitors including *Ph. infestans* Pi_EPI1_PITG_22681 - Pi_EPI9_PITG_13292, *Saprolegnia parasitica* Sp_001_01027, *A. euteiches* Ae_11AL6547, and *Plasmopara halstedii* CB175657. The conserved cysteine residues that defined the Kazal family of protease inhibitors are marked with asterisks. Interestingly, most of the putative proteins may contain multiple Kazal-like domains which are shown using the suffix _d(number).

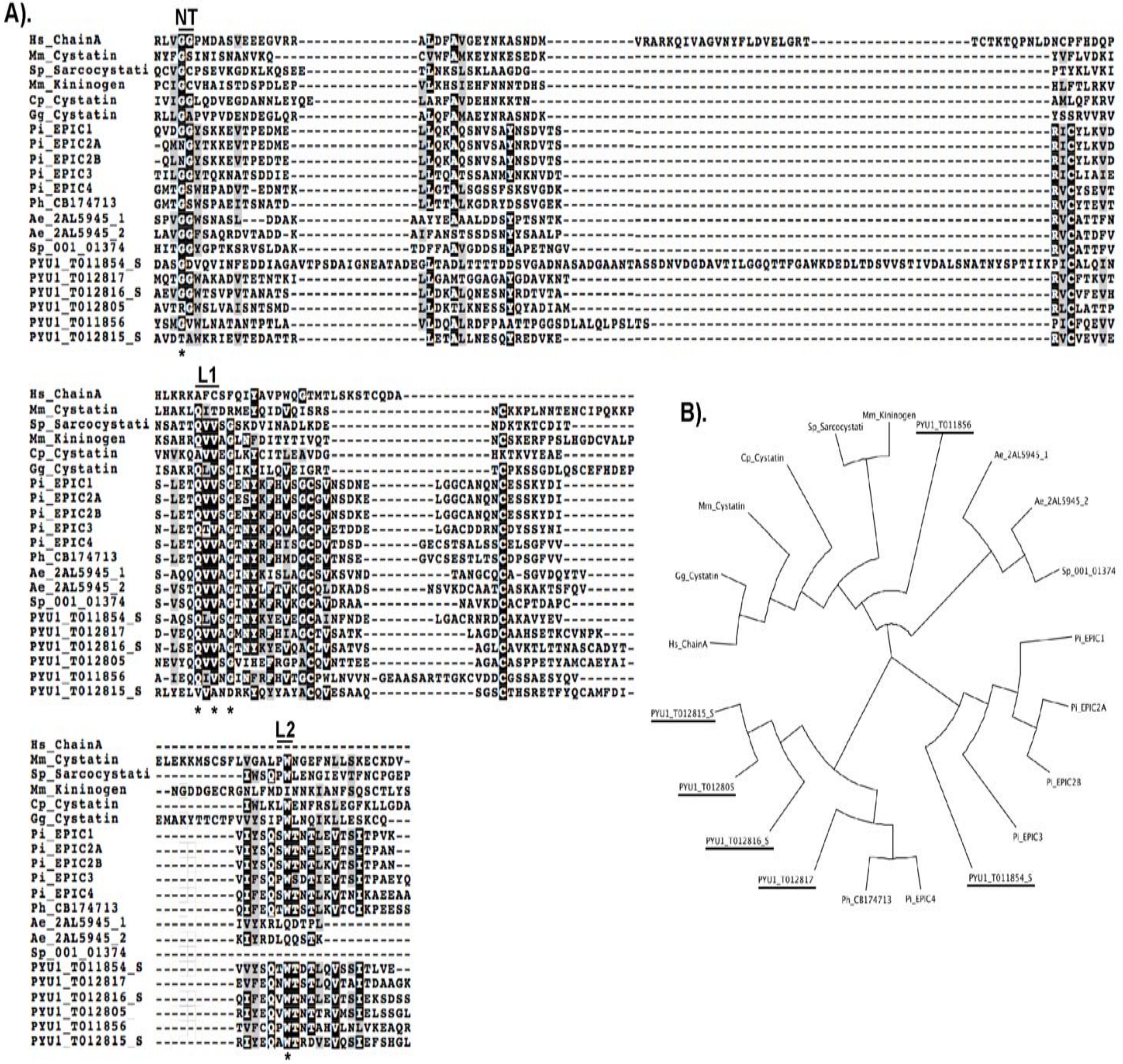

Figure S15. A family of cystatin-like protease inhibitors predicted in *P. ultimum* genome. A). Sequence alignment of six *P. ultimum* cystatin-like protease inhibitors PYU1_T011854, PYU_T012817, PYU1_T012816, PYU1_T012805, PYU1, T011856, PYU1_T011815 (_S suffix = indicates a protein sequence carrying signal peptide) with fifteen other cystatin-like protease inhibitors from various oomycetes (*Ph. infestans* Pi_EPIC1_PITG_09169 - Pi_EPIC2A_PITG_09175 - Pi_EPIC2B_PITG_09173 - Pi_EPIC3_PITG_14891 - Pi_EPI41_PITG_00058, *Plasmopara halstedii* CB174713, *A. euteiches* Ae_2AL5945, *Saprolegnia parasitica* Sp_001_01374), from plants (*Carica papaya* Cp_Cystatin gi|311505), from animals (insect *Sarcophaga peregrina* Sp_Sarcocystatin gi|399335, chicken Gg_Cystatin P01038, mouse Mm_Cystatin gi|6226846 Mm_Kininogen gi|12643495, human Hs_Chain A gi14278690). The proposed active-site residues in cystatins, including the N-terminal trunk (NT), first binding loop (L1) and second binding loop (L2) are shown. The conserved amino acids in the active site are marked with asterisks. **B).** Phylogenetic analysis of six *P. ultimum* and other fifteen cystatin-like protease inhibitors. Five out of six cystatins in *P. ultimum* are clustered in two groups with other oomycete cystatins. PYU1_T011854 appears to be the most divergent of the family. *P. ultimum* cystatins are underlined.

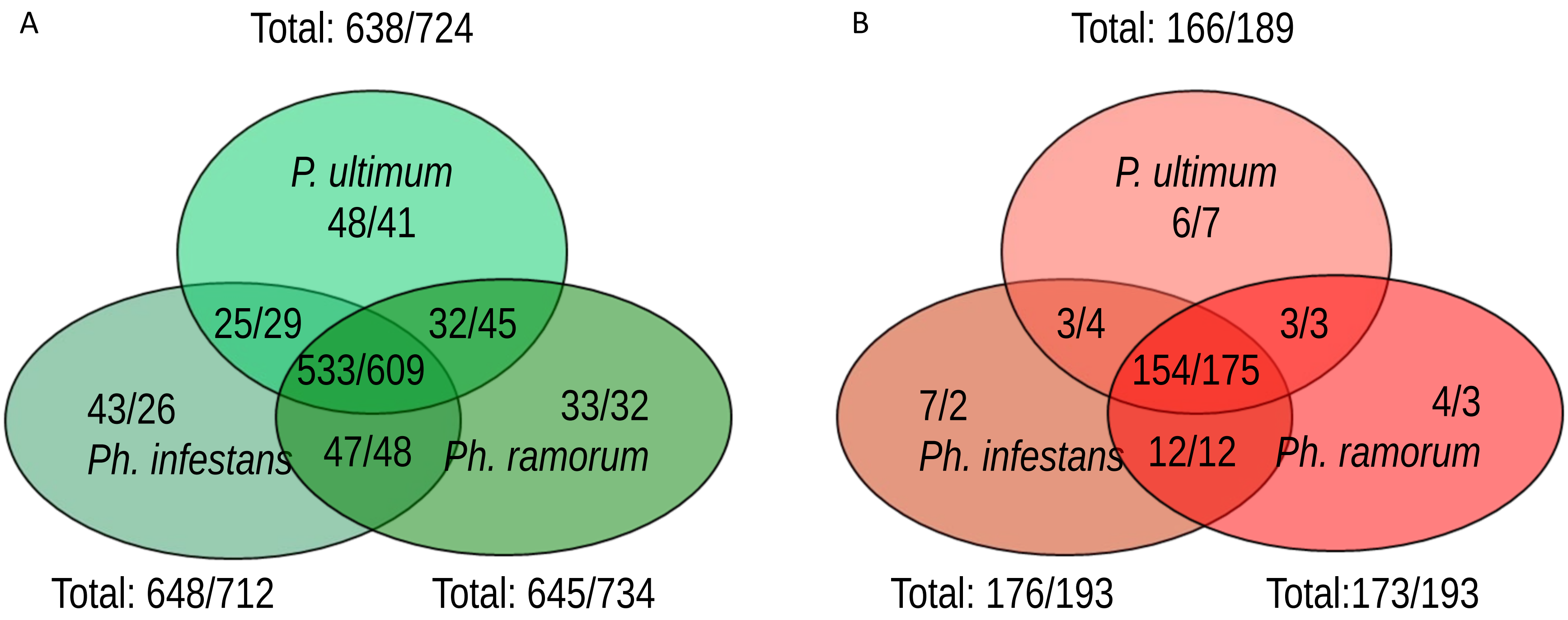

Figure S16. Venn diagram showing the homology (E-value < 10^{-5}) of the diatom genes derived from green (A) and red (B) algae according to Moustafa et al. [166] with *P. ultimum*, *Ph. infestans* and *Ph. ramorum*. The first number of each pair represents the number of matches to *Phaeodactylum* genes and the second one is the number of matches to *Thalassiosira*. The results with the other two possible Venn diagrams when comparing *Ph. sojae* were very similar.
