# Supplementary figures and images for: Genome sequence of the necrotrophic plant pathogen Pythium ultimum reveals original pathogenicity mechanisms and effector repertoire
Source: Genome Biol. 2010 Jul 13;11(7):R73. doi: 10.1186/gb-2010-11-7-r73 (PMC2926784; doi:10.1186/gb-2010-11-7-r73)

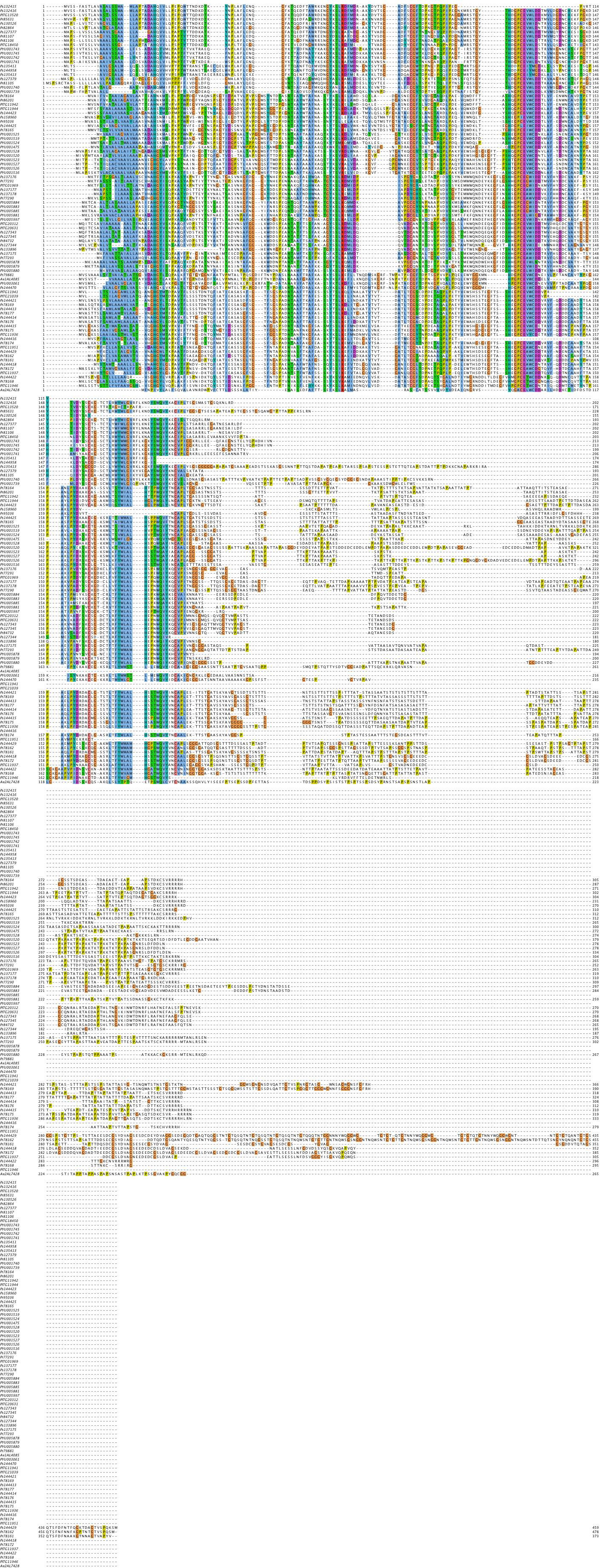

Supplement: Additional file 4 — Multiple sequence alignment of oomycete proteins with similarity to P. ultimum Family 3 proteins. Predicted secreted proteins (91) with similarity to Family 3 proteins from various oomycete species were aligned demonstrating the YxSL[KR] motif. [file gb-2010-11-7-r73-S4.png]
